# Can H_2_ be Superacidic? A Computational Study of Triel-Bonded Brønsted Acids

**DOI:** 10.1021/acs.jpca.4c02663

**Published:** 2024-06-13

**Authors:** Jakub Brzeski

**Affiliations:** Faculty of Chemistry, University of Gdańsk, Wita Stwosza 63, Gdańsk 80-308, Poland

## Abstract

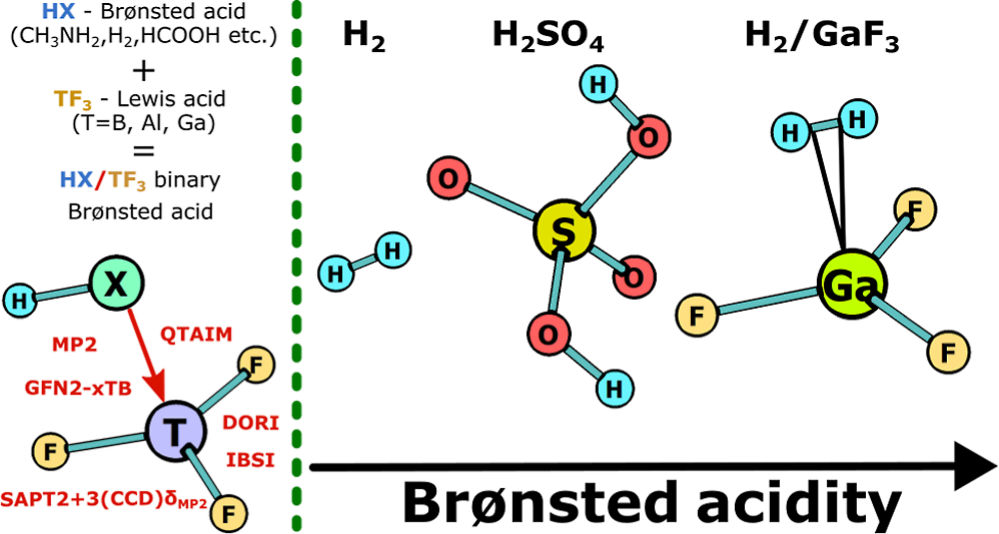

The abundance of XIII group element compounds in science and industry together with their electron-deficient character gives rise to their influence on properties of the systems they interact with. This paper is an attempt to assess the strength, nature, and effect of formation of a triel bond on acidity. A wide set of Brønsted acids among others comprising hydrocarbons, halogen hydrides, and amines bonded with B, Al, and Ga trifluorides forming HX/TF_3_ was selected for the research. Various computational approaches (e.g., MP2, GFN2-xTB, SAPT2 + 3(CCD)δ_MP2_, quantum theory of atoms in molecules analysis, and density overlap regions indicator) are used to describe the triel-bonded systems. Among other things, it was found that the electrostatics may not be the dominant contribution to the triel binding in some cases. Additionally, it was established that even weak Brønsted acids such as C_2_H_2_ or H_2_ may be superacidic if bonded to a Lewis acid (TF_3_) that is strong enough. The calculations indicate a significant covalent character of some of the studied HX/TF_3_ triel-bonded systems. Moreover, the effect of solvation of HX with TF_3_ as well as that of the reverse process on the acidity of the resulting system is thoroughly described.

## 1 Introduction

π–Holes are defined as the regions on the electrostatic potential map of a given system arising from charge depletion, i.e., positive values of electrostatic potential. The conditions that have to be met for a hole to be referred to as π and not σ are that the molecule of interest needs to be planar and that the lobe corresponding to the positive electrostatic potential has to be perpendicular to the molecular plane.^1,2^ The elements of the third main group of the periodic table are known to exhibit such properties.^3,4^ The bonds formed as an effect of the interaction between π–hole localized on the boron group element and electron density donor are referred to as triel bonds.^5−10^ Naturally, this interplay may be regarded as Lewis acid–Lewis base type interaction, where triel compounds act as Lewis acids. If the Lewis base part of the system formed this way is a Brønsted acid, then the whole system may be regarded as a Brønsted acid as well.^11^ Olah has proposed to refer to the acids thus formed as conjugate Brønsted–Lewis acids belonging to the binary acids family.^12^

The triel bond itself has received much attention in the past decade. A significant impact on our knowledge of the triel bond comes from Grabowski, who has studied triel-bonded boron halide complexes with N_2_, NH_3_, and HCN^13^ as well as aluminum-based ones.^2^ He has also delved into triel-bonded complexes of ethylene, acetylene,^14^ and benzene.^15^ Moreover, his paper devoted to characterization of the triel bonds formed by B, Al, Ga, In, and Tl in various molecular systems also needs to be mentioned here.^6^ The other important papers come from the joint effort of Li and Scheiner, who have (among other things) studied triel-bonded carbenes^16^ and compared the effects between triel and regium bond^17^ as well.^18^ As an effect of Scheiner’s collaboration with Zierkiewicz’s group, papers on the anion–anion and anion–neutral triel bonded systems^19^ as well as the comparison between intra- and intermolecular triel bonds have appeared.^20^ The other important reports that need to be mentioned here are the ones showing the anion−π triel bond interactions,^21^ describing hydride-triel bonds,^22^ and the delineating charge-assisted triel bonds in a solid state.^23^

**Figure 1:**
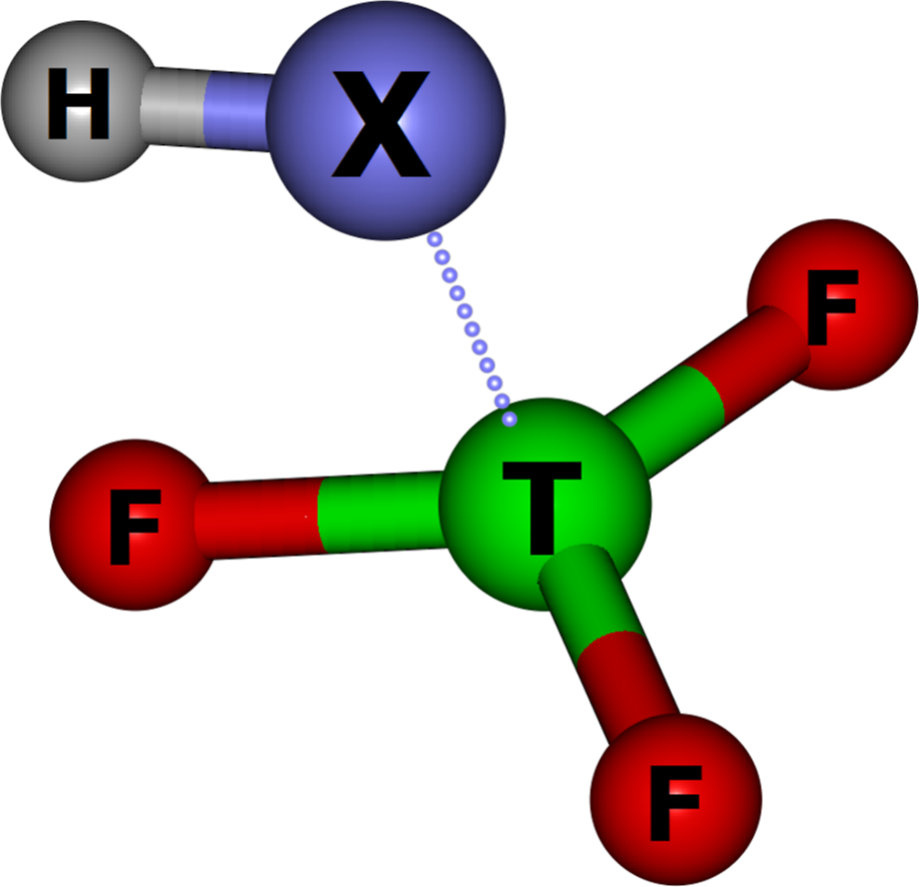
General structure of the triel-bonded binary acids studied in this paper. T stands for triel atom (B, Al, or Ga), whereas HX represents a Brønsted acid.

The influence of the formation of intramolecular interactions on the acidity was a subject of study of many papers.^24−27^ Most of the papers however limit their focus to the influence of the H-bond,^28,29^ which is understandable bearing in mind its importance and abundance in nature. It is our group that has studied the influence of the formation other types of interactions, i.e., tetrel^27^ and pnictogen^24^ on the Brønsted acidity. The triel-bonded binary Brønsted acids themselves are rather well described in the literature.^30−33^ Among others, the effect of the electron-accepting ability of the substituents in the triel part of the binary acids on the acidity of the resulting system was described by our group.^11^ It was also found in the past that the acidity of binary acids may be increased by a solvation of mineral acids (such as HClO_4_) with either AlF_3_ or SbF_5_.^34^ Additionally, our research group has also found that the formation of a triel bond between AlF_3_ and even weak Brønsted acids such as H_2_O or H_2_S leads to the formation of Brønsted superacids,^35^ i.e., acids stronger than a 100% concentrated H_2_SO_4_.^36^ Hence, although there are a number of papers describing triel-bonded acids, to the best of the authors’ knowledge, there are none trying to associate the strength and nature of the said bond with Brønsted acidity. This paper seeks to address this issue by thoroughly examining the TF_3_-bonded (vide Figure 1) acids with the use of various computational methods. Besides, it tries to comprehensively describe the triel interactions formed by studied HX/TF_3_ acids.

## 2 Methods

The equilibrium geometries of HX/TF_3_ (where HX = (CH_3_)_2_NH, CH_3_NH_2_, NH_3_, C_2_H_4_, C_2_H_2_, H_2_, H_2_O, CH_3_OH, HCOOH, CH_3_COOH, HF, HCl and T = B, Al, Ga) triel-bonded binary acids as well as their corresponding bases (formed after deprotonation) were established using the Møller–Plesset MP2 method^37,38^ (with core orbitals frozen). Two Dunning type basis sets were used during the calculations, namely, the aug-cc-pV(T+d)Z^39,40^ basis set with an additional tight d function was used for triel atoms, whereas a regular aug-cc-pVTZ^41,42^ was used for all of the remaining atoms. Subsequently, the vibrational frequencies were calculated for the mentioned systems to confirm that they are true minima on the potential energy surfaces. These calculations were performed using the Gaussian 16 (Rev. C.01) package.^43^

The acidities of the studied systems were assessed based on the Gibbs free energies of the deprotonation process (). In order to determine the impact of triel bonding formation on the acidity of the resulting HX/TF_3_ systems, the values of  calculated for binary acids were compared to that of the sole HX acids resulting in . The natural bond orbital (NBO 6.0)^44^ analysis was performed using MP2 densities in order to determine the charge transfer associated with the formation of the binary acids.

The electron density topology analysis, as implemented in Bader’s quantum theory of atoms in molecules analysis (QTAIM), was employed in order to characterize the interactions between AlF_3_ and HX.^45^ For the study of molecular interactions, the so-called bond critical points (BCPs), i.e., points where the two eigenvalues of the Hessian matrix of electron density ρ are negative between two atoms, are scrutinized.^46^ The values of the Laplacian of the electron density at a given BCP (∇^2^ρ_BCP_) are used to differentiate between closed- and open-shell interactions. Namely, negative values of the Laplacian indicate an open-shell interaction, whereas when the Laplacian takes a positive value, then the interaction is regarded as a closed-shell one. The opened/closed classification has been further improved by Bianchi et al., who have proposed a classification based on the potential to kinetic energy density ratio—|*V*_BCP_|/*G*_BCP_.^47^ Based on their division, the interactions may be classified as follows
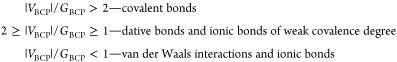


The concept of a bond degree (BD = *H*_BCP_/ρ_BCP_) may also be used to assess the degree of covalency of a given interaction.^48^ Namely, for the values of |*V*_BCP_|/*G*_BCP_ lower than 1, *H*_BCP_/ρ_BCP_ is positive and the larger values it takes the more closed in nature and weaker the interaction. On the contrary, for an interaction characterized by |*V*_BCP_|/*G*_BCP_ values higher than 1, the *H*_BCP_/ρ_BCP_ is negative and the higher its absolute value the more covalent the interaction.

The HX/TF_3_ interaction energy was calculated in a few manners, namely, via the supermolecular approach of Boys and Bernardi^49^ with the use of MP2 and through the SAPT^50^ method. All the calculations mentioned in this paragraph were performed using the aTZ basis sets mentioned earlier. In the supermolecular approach, the interaction energy is calculated taking the basis set superposition error into account as
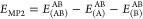


In the above formula, superscripts represent the basis set used for the calculation, whereas subscripts denote the system for which the energy is calculated. In the next step, the TF_3_ deformation upon binding with HX was calculated
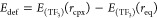
as a difference between its energy in the structure it exhibits in the complex (*r*_cpx_) and that of the equilibrium geometry (*r*_eq_). Finally, the HX/TF_3_ binding energy was calculated as follows
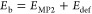


Symmetry-adapted-perturbation-theory (SAPT) calculations were performed in order to both assess the binding energy between the units forming binary acids and to determine the contribution of each component, i.e., electrostatics, exchange, induction, and dispersion to the total interaction energy. In the SAPT approach, the total Hamiltonian of the system is expressed as the sum of the monomer Fock operators (*F̂*_*X*_), intramonomer correlation operators (*Ŵ*_*X*_) and interaction potential operator (*V̂*_*XY*_)
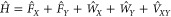


Due to the relatively small size of the considered systems, we have decided to apply the most sophisticated SAPT method available in PSI4 package—SAPT2 + 3(CCD)δ_MP2_. This accounts for CC dispersion and MP2 induction correction. The stabilization energy (*E*_stab_) of all HX/TF_3_ complexes due to the charge transfer calculated at the SAPT2 + 3(CCD) level of theory was extracted from the induction energy method as proposed by Stone and Misquitta.^51^ All SAPT^52^ calculations were carried out using PSI4 (1.8 release) software.^53^

The density overlap regions indicator (DORI)^54^ method was used to visually characterize the interactions between selected HX and TF_3_ and the process of the dissociation of selected binary acids. The DORI [γ(**r**)] is defined as
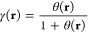
where θ(**r**) = (∇(∇ρ(**r**)/ρ(**r**))^2^)^2^/(∇ρ(**r**)/(ρ(**r**)))^6^.^55^ This method allows one to simultaneously describe both covalent as well as the noncovalent interactions. In order to differentiate between bonding and nonbonding interactions within studied systems, the γ(**r**) isosurfaces were colored with the use of sgn(λ_2_)ρ(**r**) to get an easy-to-understand picture. Here, λ_2_ is a second eigenvalue of the Laplacian of the electron density, which is λ_2_ < 0 for bonding and λ_2_ > 0 for nonbonding interactions. The method itself has been successfully used to describe the interactions occurring within the various molecular systems.^54,56^ For the sake of comparison, the other method of visual analysis of interactions, an interaction region indicator (IRI), was employed.^57^ For a standard calculation, IRI is defined as the gradient norm of ρ(**r**) weighted by scaled electron density factor—[ρ(**r**)]^*a*^, with *a* = 1.1. The IRI analyses have solely confirmed DORI results. For this reason, the outcomes of the former are not reported here.

The intrinsic bond strength index (IBSI) characterizing triel bonds within studied HX/TF_3_ systems was calculated using both the independent gradient model (IGM) and its δ*g* parameter. IGM describes the noninteracting system of reference, whereas δ*g* quantifies the electron sharing between two interacting fragments.^58,59^ Altogether, the expression for dimensionless IBSI normalized to one for a H_2_ molecule is given by
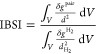
where δ*g* = |∇ρ^IGM^| – |∇ρ|, whereas *d* is the distance between two interacting atoms. For the hydrogen and halogen–covalent interactions studied by Klein et al.,^200^ it was found that IBSI takes values in the range from 0.00 to 0.15. Nonetheless, it is worth noting here that triel interactions are generally stronger than hydrogen or halogen, mainly due to the inherently electron-deficient character of XIII group elements. For a three-centered interaction observed in HX/TF_3_ where HX = H_2_, C_2_H_2_, or C_2_H_4_, a mean value of the IBSI indices is reported here. However, it should be stated that the individual values are close to each other in every example. The QTAIM, DORI, IRI, and IGM analyses were all performed using the wave function analysis package—Multiwfn (ver. 3.8).^60^

Finally, to assess the influence of stoichiometry of the conjugate systems on the acidity, the calculations on (HCOOH)_*n*_/(AlF_3_)_6–*n*_ (*n* = 1–5) model systems were conducted. In the first step, the calculations employing the conformer–rotamer ensemble sampling tool (CREST-version 2.11)^61^ software were used. Namely, the iterative version of the meta-dynamics genetic structure crossing algorithms (iMTD-GC)^62^ workflow was used to find the low lying conformers of all (HCOOH)_*n*_/(AlF_3_)_6–*n*_ systems and their corresponding bases formed after deprotonation. The calculations were carried out at the GFN2-xTB (including D4 dispersion) level of theory.^63^ The lowest in terms of energy isomer in each stoichiometry was then subjected to optimization and vibrational frequency calculation at the MP2/aug-cc-pV(D+d)Z (Al)/aug-cc-pVDZ (remaining atoms) level of theory.

## 3 Results

### 3.1 Equilibrium Structures of Binary Acids

**Figure 2:**
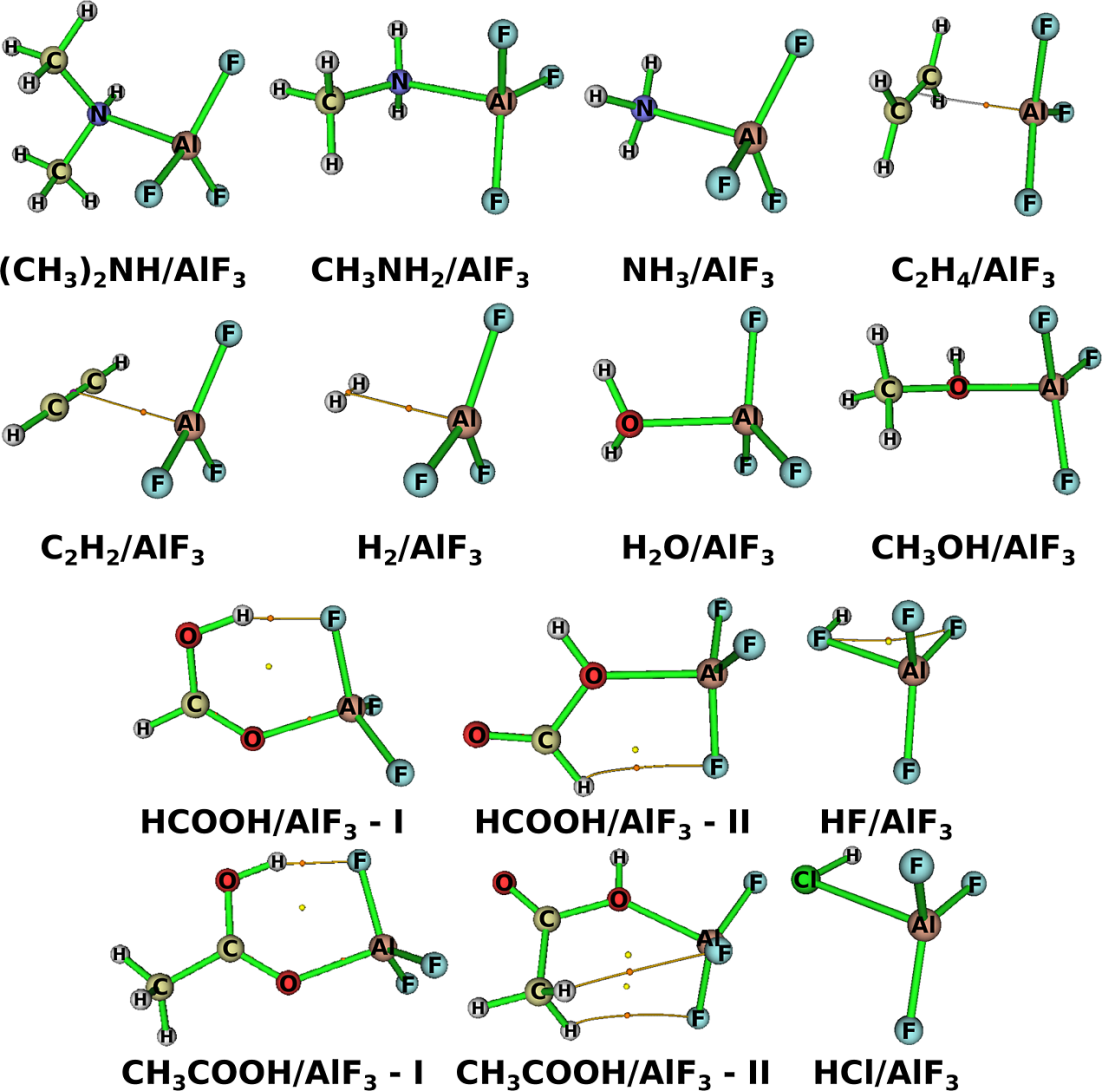
QTAIM molecular graphs of HX/AlF_3_ triel-bonded binary acids. Critical points together with corresponding bond paths are represented by dots and lines, respectively. Green lines correspond to covalent and strong noncovalent interactions, whereas the remaining lines denote the weak noncovalent character of the interaction. Bond and ring critical points are depicted with orange and yellow dots, respectively.

The equilibrium structures of triel-bonded acids studied in this paper consist of a Lewis acid (TF_3_) unit tethered to a Lewis base (HX) unit. The equilibrium structures of AlF_3_-bonded acids are depicted in Figure 2. The corresponding equilibrium structures of BF_3_ and GaF_3_ are analogous to those observed for AlF_3_. Hence, we have decided to depict only those corresponding to AlF_3_-bonded acids in the manuscript. Nonetheless, structures of all acids studied in this paper together with QTAIM analysis results are presented in Table S1.

The equilibrium structures of triel-bonded binary acids are foreseeable and consist of a Lewis base (HX) tethered to a Lewis acid (TF_3_) via triel bonds spanning from 1.642 for CH_3_COOH/BF_3_-I to 2.931 Å for C_2_H_2_/BF_3._ As described in succeeding paragraphs, a triel bond may be assisted (or even replaced) by other types of interaction. The triel bond lengths seem to follow the general trend that they are inversely proportional to the basicity of the Lewis base, e.g., CH_3_NH_2_/TF_3_ bond lengths span from 1.651 to 1.997 Å, whereas that of C_2_H_2_ falls in between 2.307 and 2.931 Å.

The nature of interactions between HX and TF_3_ depends on the structure of HX and TF_3_. Namely, besides the triel interaction, hydrogen and even halogen interactions are present in some cases. For the cases where (CH_3_)_2_NH, CH_3_NH_2_, NH_3_, C_2_H_4_, C_2_H_2_, H_2_, H_2_O, or CH_3_OH was used as electron density donors, only triel interactions were calculated via QTAIM analysis, regardless of the TF_3_ considered. This makes the binary acids formed by said Brønsted acids entirely triel-bonded.

Hydrogen interactions are present in cases when carboxylic acids (HCOOH or CH_3_COOH) are used as Lewis bases. The presence of two donor atoms in the said systems gives rise to the formation of two isomeric forms of HCOOH/TF_3_ and CH_3_COOH/TF_3_ (depicted in Figure 2). In the lower energy isomer (RCOOH/TF_3_-I), the carboxylic group acts as both a hydrogen and triel bond donor. Namely, the −OH part of −COOH gives a rise to a H-bond, whereas the remaining =O donates its density to form a triel bond with a TF_3_ unit. In the higher energy isomer (RCOOH/TF_3_-II), the −OH part of the carboxylic moiety engages in the formation of a triel bond, whereas the H-bond arises from the interaction of H atoms of R in RCOOH with fluorine atoms of TF_3_. The relative energy of the discussed isomers (see Table 1) rise with the atomic number of T. Even for the lightest of triel atoms, the difference is significant enough to ensure its dominance in the RCOOH/BF_3_ population in standard conditions.

**Table 1:** Relative Electronic Energies—Δ*E* (in kcal/mol) of RCOOH/TF_3_ Isomers

	Δ*E*	
T	HCOOH	CH_3_COOH
B	7.44	8.77
Al	15.40	14.26
Ga	16.34	14.79

The ca. 15 kcal/mol energies calculated for Al and Ga bonded acids indicate that the Lewis acidities of AlF_3_ and GaF_3_ are similar. This concurs well with the fluoride ion affinities (FIAs) obtained [at CCSD(T)/CBS level] for the discussed species by Erdmann et al.,^64^ who have calculated the FIAs of BF_3_, AlF_3_, and GaF_3_ to be equal to 84.2, 115.5, and 108.6 kcal/mol, respectively. The differences in energies arise from the different electron-accepting/-donating abilities of −OH, =O and –CH groups.

**Figure 3:**
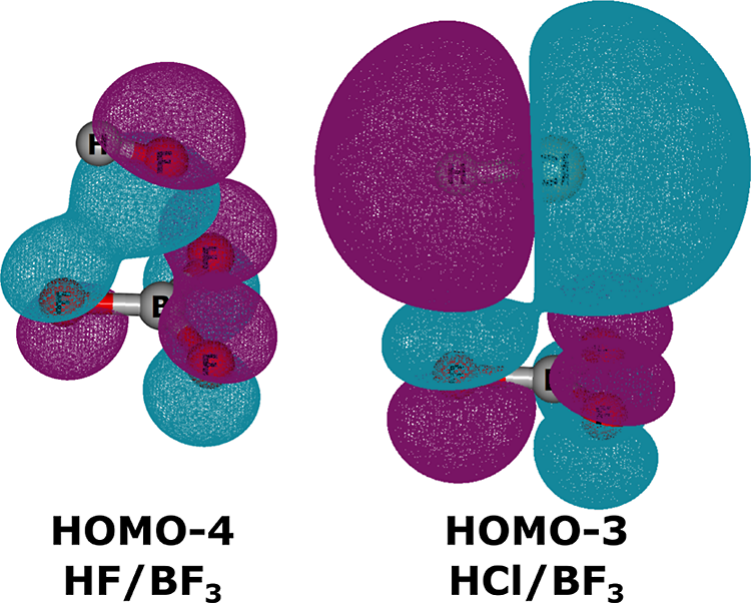
Plots of the selected molecular orbitals of HF/BF_3_ and HCl/BF_3_ confirm that said binary acids are in fact halogen-bonded.

The other type of noncovalent interaction present in some of the studied binary acids is the halogen bond. The meaning of this interaction seems to diminish with the atom number of the triel center. Namely, it is extremely important for BF_3_-bonded acids to the degree that QTAIM analysis allows for the detection of halogen, i.e., H···F, O···F, F···F, or Cl···F for some of the BF_3_-bonded acids. No triel interaction was found during the QTAIM analysis of H_2_/BF_3_, HCOOH/BF_3_-II, and HCl/BF_3_. In the case of HF/AlF_3_ and HCl/AlF_3_, two kinds of interactions, namely, triel and halogen, were identified, whereas for GaF_3_-bonded counterparts, only triel interactions are observed. The plots of selected molecular orbitals of HF/BF_3_ and HCl/BF_3_ are another proof of the halogen nature of interaction between HF/HCl and BF_3_. As is evident from Figure 3 the greatest contribution to both depicted molecular orbitals comes from the p type atomic orbitals of halogen atoms. The portrayed molecular orbitals clearly indicate a bonding interaction between two halogen atoms in HX and TF_3_.

The reasons due to which the interaction within H_2_/BF_3_ may be referred to as halogen and not hydrogen are that QTAIM analysis (see Table S1) reveals an interaction between the H atom of H_2_ and F atom of BF_3_ and that the NBO analysis indicates the charge transfer from H_2_ to BF_3_ (see Table 2). Nonetheless, it is important to realize that the 0.001*e* NBO charge transfer is far too small for the interaction to be categorized with certainty.

**Table 2:** Parameters Describing the HX/TF_3_ Interactions: Bond Lengths (in Å), NBO Charge Transfer from HX to TF_3_ (in *e*), Electron Density (ρ_BCP_), Laplacian Thereof (∇^2^ρ_BCP_) and *H*_BCP_/ρ_BCP_ in au, Whereas |*V*_BCP_|/*G*_BCP_ and IBSI Are Dimensionless Parameters^a^

system	*R*	NBO	ρ_BCP_	∇^2^ρ_BCP_	|*V*_BCP_|/*G*_BCP_	H_BCP_/ρ_BCP_	IBSI
(CH_3_)_2_NH/BF_3_	1.651	0.335	0.121	0.292	1.56	–0.78	0.2765
CH3NH2/BF3	1.988	0.341	0.117	0.311	1.53	–0.76	0.2679
NH3/BF3	1.653	0.334	0.109	0.319	1.49	–0.71	0.2494
C2H4/BF3	2.918	0.007	0.009	0.026	0.86	0.08	0.0117
C2H2/BF3	2.931	0.006	0.009	0.027	0.82	0.12	0.0117
H2/BF3	2.754	0.002	0.005	0.020	0.73	0.21	0.0058
H2O/BF3	1.782	0.204	0.063	0.253	1.33	–0.49	0.1485
CH3OH/BF3	1.685	0.241	0.082	0.373	1.31	–0.52	0.1975
HCOOH/BF3-I	1.674	0.228	0.083	0.379	1.31	–0.52	0.2040
	1.633		0.044	0.165	1.10	–0.11	
HCOOH/BF3-II	1.963	0.019	0.015	0.060	0.88	0.10	0.0273
CH3COOH/BF3-I	1.641	0.242	0.091	0.421	1.32	–0.54	0.2247
	1.616		0.046	0.169	1.12	–0.13	
CH3COOH/BF3-II	2.301	0.030	0.019	0.067	1.00	0.00	0.0371
b	2.735		0.005	0.021	0.76	0.22	
HF/BF3^c^	2.775	0.014	0.012	0.053	0.82	0.17	0.0193
HCl/BF3^c^	3.270	0.011	0.008	0.029	0.80	0.15	0.0119
(CH3)2NH/AlF3	1.985	0.105	0.064	0.364	1.04	–0.06	0.1019
CH3NH2/AlF3	1.988	0.117	0.063	0.359	1.03	–0.04	0.0972
NH3/AlF3	2.000	0.126	0.052	0.403	0.90	0.18	0.0900
C2H4/AlF3	2.376	0.094	0.029	0.098	1.08	–0.07	0.0515
C2H2/AlF3	2.360	0.079	0.029	0.112	1.03	–0.03	0.0548
H2/AlF3	2.105	0.053	0.018	0.086	0.91	0.11	0.0349
H2O/AlF3	1.944	0.103	0.052	0.403	0.90	0.18	0.0790
CH3OH/AlF3	1.915	0.098	0.057	0.442	0.92	0.14	0.0888
HCOOH/AlF3-I	1.894	0.064	0.061	0.467	0.93	0.12	0.0941
	1.547		0.055	0.170	1.22	–0.22	
HCOOH/AlF3-II	1.963	0.085	0.047	0.374	0.87	0.22	0.0765
	2.327		0.012	0.054	0.80	0.20	
CH3COOH/AlF3-I	1.878	0.068	0.064	0.489	0.94	0.10	0.0978
	1.555		0.055	0.170	1.22	–0.22	
CH3COOH/AlF3-II	1.941	0.089	0.051	0.402	0.89	0.20	0.0827
b	2.552		0.007	0.030	0.80	0.17	
HF/AlF3	1.989	0.080	0.035	0.313	0.79	0.38	0.0610
F–F interaction	2.517		0.026	0.104	1.03	–0.03	
HCl/AlF3	2.491	0.130	0.026	0.124	0.95	0.06	0.0488
(CH3)2NH/GaF3	1.997	0.174	0.094	0.358	1.27	–0.36	0.1439
CH3NH2/GaF3	2.002	0.180	0.092	0.355	1.26	–0.34	0.1369
NH3/GaF3	2.014	0.182	0.087	0.343	1.25	–0.33	0.1280
C2H4/GaF3	2.292	0.145	0.050	0.098	1.36	–0.27	0.0690
C2H2/GaF3	2.307	0.120	0.048	0.118	1.28	–0.24	0.0692
H2/GaF3	2.075	0.063	0.031	0.108	1.09	–0.08	0.0420
H2O/GaF3	2.000	0.131	0.074	0.404	1.13	–0.20	0.1027
CH3OH/GaF3	1.974	0.133	0.080	0.438	1.14	–0.22	0.1149
HCOOH/GaF3-I	1.932	0.088	0.086	0.469	1.15	–0.24	0.1272
	1.508		0.063	0.179	1.28	–0.28	
HCOOH/GaF3-II	2.017	0.106	0.068	0.383	1.10	–0.16	0.0970
	2.315		0.012	0.053	0.79	0.20	
CH3COOH/GaF3-I	1.932	0.096	0.089	0.491	1.15	–0.25	0.1333
	1.508		0.061	0.177	1.27	–0.27	
CH3COOH/GaF3-II	1.993	0.112	0.073	0.412	1.11	–0.18	0.1060
b	2.517		0.008	0.032	0.80	0.17	
HF/GaF3	2.059	0.087	0.051	0.334	1.01	–0.01	0.0721
HCl/GaF3	2.486	0.149	0.041	0.133	1.18	–0.17	0.0611

a Rows of data containing the formula of the binary acid describe the triel bonds, which (if those exist) are followed by those characterizing H-bonds.

b There are two H-bond interactions characterized by the same set of numbers in the equilibrium structure of the triel-bonded acid.

c Although the orientation of molecules forming HF/BF_3_ and HCl/BF_3_ suggests the existence of triel interaction, the QTAIM analysis does not allow for the detection of one. Numbers in the Table describe the F···F and Cl···F interaction for HF/BF_3_ and HCl/BF_3_, respectively.

The H_2_ molecule and studied hydrocarbons are bound to TF_3_ in a η^2^ mode, in the same manner that was observed for H_2_ interactions with carborane and caralumane Lewis acids.^65^

### 3.2 Analyses Based on Electron Density

As mentioned in the prior paragraphs of the manuscript, QTAIM and NBO analyses were carried out in order to characterize the interactions between HX and TF_3_ in the studied binary acids. The parameters describing these calculations are collected in Table 2. The amount of the electron density transfer associated with the formation of HX/TF_3_ binary acids may be assessed using the NBO analysis. The values presented in Table 2 indicate that the charge transfer between the HX and TF_3_ units is proportional to the Lewis basicity of the former. On another note, one would expect that the charge transfer would be comparative with the relative Lewis acidities (as, for example, expressed in FIAs) of the studied TF_3_ compounds. According to the finding by Erdmann et al.,^64^ the said acidity decreases in the following order: AlF_3_ > GaF_3_ > BF_3_. The highest charge transfer of 0.325*e* was calculated for (CH_3_)_2_NH/BF_3_ and CH_3_NH_2_/BF_3_, whereas the lowest equal to 0.005*e* was found for H_2_/BF_3_. It can be concluded that the charge transfer to GaF_3_ is always greater than that to AlF_3_ regardless of the HX considered. The situation is more complicated when it comes to the comparison with BF_3_, as it accepts more electron density than its AlF_3_ and GaF_3_ counterparts for complexes with relatively strong Lewis bases such as amines, ammonia, water, methanol, and carboxylic acids (via RCOOH/TF_3_-I binding mode). In the cases where weak Lewis bases such as hydrogen molecule, ethyne, or hydrogen fluoride are used, HX, AlF_3_, and GaF_3_ tend to accept more electron density than BF_3_.

The triel interactions within the systems studied here exhibit a wide range of lengths (*R*), as the shortest distance of 1.641 Å was found for CH_3_COOH/BF_3_-I whereas the longest interaction of 3.270 Å was calculated for HCl/BF_3_.

The trends observed for NBO charge transfer are rather similar to those observed from the ρ_BCP_ of the corresponding triel interaction. First, surprisingly, both top seven and bottom seven compounds characterized by both the highest and lowest values of NBO charge transfer and ρ_BCP_ are BF_3_-bonded. This is an indicative of a peculiar character of the boron atom itself. Second, the electron density calculated for triel-bonded complexes as well as the NBO charge-transferred characterizing GaF_3_-bonded systems are always higher than that determined for AlF_3_. Similarly, the higher affinity of BF_3_ toward strong Lewis bases than that calculated for AlF_3_ and GaF_3_ may also be observed from the calculated ρ_BCP_ and NBO values. It is also worth noting here that the ρ_BCP_ values corresponding to the triel interactions are always higher than those calculated for a neighboring hydrogen bond. For instance, the triel bond between the O atom of HCOOH and B atom of BF_3_ in HCOOH/BF_3_-I is characterized by a value of ρ_BCP_ equal to 0.083 au, whereas that of an adjacent hydrogen bond is characterized by a nearly two times smaller value of 0.044 au. When it comes to the Laplacian of the electron density of triel interaction (∇^2^ρ_BCP_), all values turned out to be positive, indicating the (as anticipated) closed-shell nature of the interaction. Values of the ∇^2^ρ_BCP_ calculated for BF_3_-bonded compounds turned out to be smaller than their corresponding AlF_3_ and GaF_3_ counterparts, suggesting weaker bonding in the former.

As can be seen from the |*V*_BCP_|/*G*_BCP_ values presented in Table 2, the interactions between HX and TF_3_ may be categorized as either dative of weak covalence degree (values ≥ 1) or mainly van der Waals-based (values < 1). The highest values of the discussed ratio, which are close to 1.50, were calculated for the BF_3_-bonded complexes of ammonia and amines, indicating the strong dative nature of the interaction. Unsurprisingly, the lowest value of 0.73 was found for H_2_/BF_3_. No straightforward trend is observed between the value of |*V*_BCP_|/*G*_BCP_ and the Lewis acidity of TF_3_ compounds used in this study, as the calculated values of |*V*_BCP_|/*G*_BCP_ seem to be highly dependent on both type of triel atom as well as the nature and structure of the HX moiety. The values of *H*_BCP_/ρ_BCP_ ratio clearly demonstrate that the triel bonds formed between BF_3_ or GaF_3_ and amines or ammonia exhibit a significant covalent character. Once more, the situation is different when AF_3_ is employed as an electron density acceptor, as the corresponding *H*_BCP_/ρ_BCP_ values are all close to zero. The highest values of discussed parameter, i.e., the most closed-shell of the interaction, was calculated for H_2_/BF_3_ (*H*_BCP_/ρ_BCP_ = 0.21).

When it comes to IBSI indices, we have found that out of 42 studied systems, IBSI is lower than 0.15 for 36 of them. Interestingly, all of the 6 acids characterized by the highest values of IBSI are BF_3_-based. Said acids are that of H_2_O, CH_3_OH, HCOOH (-I), CH_3_COOH (-I), NH_3_, CH_3_NH_2_, and (CH_3_)_2_NH/BF_3_, with corresponding values of 0.1975, 0.2040, 0.2247, 0.2494, 0.2679, and 0.2765. All of the mentioned HX Brønsted acids donors are bound with BF_3_ via atoms possessing strong electron donation properties.

Additionally, the triel bonds for binary acids of H_2_, C_2_H_2_, C_2_H_4_ HCl, HF, and HCOOH (-II) and BF_3_ are characterized by the lowest values of the IBSI index. A similar trend was observed for the NBO charge transfer. Bearing this in mind, we have decided to plot the IBSI(NBO) dependence, which is depicted in Figure S1. It is evident from the graph that there is a significant correlation between the two quantities. Nonetheless, it has to be noted here that the two cannot correlate to a much higher degree than that in the present work (*R*^2^ = 0.872) as they measure different things. Namely, NBO charge transfer was calculated as an increase of the total charge on the whole TF_3_ moiety upon complex formation, whereas IBSI describes the X–T interaction only.

### 3.3 Interaction Energies and Acidities

The binary acids studied in this paper exhibit a wide range of total interaction energies spanning from −57.41 to −0.67 and −56.98 to −0.83 in terms of *E*_MP2_ and *E*_SAPT2+3(CCD)δMP2_, respectively. The stronger the Lewis basicity of HX, the stronger the interaction between HX and TF_3_. The values of MP2 interaction energies (*E*_MP2_) are rather similar to those obtained at the *E*_SAPT2+3(CCD)δMP2_ level, as the differences do not exceed 1 kcal/mol in most examples. The highest discrepancies are observed for CH_3_COOH/GaF_3_-I, HCOOH/AlF_3_-I, NH_3_/BF_3_, and CH_3_COOH/AlF_3_-I systems, for which *E*_SAPT2+3(CCD)δMP2_ leads to the results higher by 2.15, 3.09, 3.30, and 3.45 kcal/mol than *E*_MP2_, respectively.

The values of both *E*_MP2_ and *E*_SAPT2+3(CCD)δMP2_ indicate that the interaction between HX’s like (CH_3_)_2_NH, CH_3_NH_2_, NH_3_, H_2_O, CH_3_OH, HCOOH, CH_3_COOH, and TF_3_ is always a strong one, as the value for all but two binary acids, which are not global minima on the corresponding potential energy surfaces (HCOOH/BF_3_-II and CH_3_COOH/BF_3_-II), exceeds 20 kcal/mol in every example. The deformation of TF_3_ caused by the formation of HX/TF_3_–*E*_def_ leads to destabilization of the complex. As expected, the greatest deformation energies were calculated for binary acids based on dimethylamine, methylamine, and ammonia. On the other side of the spectrum are the complexes of H_2_, which have caused a 0.01, 0.71, and 0.68 kcal/mol deformation upon binding with BF_3_, AlF_3_, and GaF_3_, respectively. Surprisingly, C_2_H_4_ and C_2_H_2_ cause similar (in terms of energetics) deformation of AlF_3_ and GaF_3_ as H_2_O or CH_3_OH. That is not the case for BF_3_, as weakly interacting C_2_H_4_ and C_2_H_2_ (−3.35 and −3.08 kcal/mol respectively) deform BF_3_ by 0.30 and 0.22 kcal/mol, respectively. Taking TF_3_ deformation energy into consideration does not change the trends observed for interaction energy. Namely, the dimethylamine-based acids are characterized by the highest absolute value of binding energy, whereas that of hydrogen are described by the lowest values of *E*_b_.

It can be said that the interaction energy in triel-bonded acids is generally higher than that observed for tetrel- and pnictogen-bonded acids.^24,27^ The individual contributions to said energy (induction, electrostatics, exchange, and dispersion) together with corresponding percent contribution describing each attractive interactions are listed in Table S3. The interaction between HX and TF_3_ is dominated by electrostatics in all but two binary acids. Previously mentioned acids are H_2_/BF_3_ and H_2_/AlF_3_. The triel interaction in H_2_/BF_3_ acid is dominated by a dispersion term as the dispersion to electrostatics ratio is equal to 1.19. On the other hand, it can be said that the H_2_/AlF_3_ acid is induction-bonded as the induction to electrostatics ratio in this case is equal to 1.15. Finally, the noncovalent interaction in H_2_/GaF_3_ acid is dominated by electrostatics term. One can thus say that the overall character of the H_2_/TF_3_ interaction changes with the atomic number of the triel atom. The significant electrostatic character of each of the triel-bonded acids accounts for at least 37.36% (for H_2_/AlF_3_) of total attractive interaction. Electrostatics has the highest attractive contribution to all the triel interactions considered. With the average of 55.87%, the contribution is common for electrostatics to exceed 60% of total contribution to interaction energy. This is especially the case for binary acids where amines or ammonia are used as a HX. This is of no surprise bearing in mind the nucleophilic character of the mentioned systems. The average contribution of induction to the attractive interactions within systems studied in this paper equal 27.36% with the highest values of 42.92, 38.49, and 35.81% calculated for H_2_/AlF_3_, HCl/AlF_3_, and CH_3_COOH/BF_3_-I, respectively. When it comes to dispersion, the average contribution was calculated to be equal to 16.78%, the value somewhat inflated by 46.95 calculated for H_2_/BF_3_. It can be noticed that of all compounds considered, dispersion interactions play the most crucial role for HX/BF_3_ systems. The values of stabilization energy due to charge transfer (*E*_stab_) vary from −141.24 kcal/mol for CH_3_NH_2_/GaF_3_ to −0.15 kcal/mol for H_2_/BF_3_. Unsurprisingly, compounds comprising a strong electron density acceptor—GaF_3_ and strong electron density donors, i.e., HX containing N or O atoms, turned out to be stabilized by the charge transfer by the highest extent. The other side of the spectrum is dominated by BF_3_-bonded acids of weak electron density donor properties such as H_2_, HCl, or HF. The significant *E*_stab_ equal to −73.73 calculated for C_2_H_4_/GaF_3_ is somewhat peculiar. This is especially distinct bearing in mind the −10.36 and −1.06 kcal/mol values calculated for C_2_H_4_/AlF_3_ and C_2_H_4_/BF_3_, respectively. The phenomenon was also observed during the NBO analysis, as the calculated value of charge transfer equal to 0.148 e within C_2_H_4_/GaF_3_ was the highest among all HX/TF_3_ containing no electronegative atoms in the HX moiety. Similarly, formation of C_2_H_4_/GaF_3_ leads to 33° deformation from planarity in GaF_3_. It appears that for the considered compounds, the stabilization of the HX/TF_3_ systems due to the charge transfer is more dependent on the nature of TF_3_ than that of HX.

As regards the influence of the formation of triel bond on acidity, it can be seen from Table 3 that it leads to the increase of acidity (decrease of ) in every studied example. Bearing in mind that the value of  equal to 303 kcal/mol corresponding to H_2_SO_4_ is a line dividing regular acids in superacids,^30^ 23 out of studied 39 may be regarded as Brønsted superacids. Amidst those are unexpected systems made of weak Brønsted acids such as C_2_H_4_/GaF_3_, C_2_H_2_/AlF_3_, or H_2_/GaF_3_. This allows us to draw a conclusion that every H-containing molecule can act as a Brønsted superacid if attached to a Lewis acid strong enough. Out of all studied compounds, those formed by HF/HCl and AlF_3_/GaF_3_ turned out to be most acidic, with  values below 270 kcal/mol (260.6 kcal/mol for the strongest acid—HCl/GaF_3_). These values agree well with findings reported in previous papers.^11^ The second most acidic group consists of carboxylic acids bonded to either AlF_3_ or GaF_3_ via the hydroxyl group. The corresponding  are however understated by the fact that the RCOOH-II/AlF_3_ or RCOOH-II/GaF_3_ equilibrium geometries are not global minima (vide Table 1). Next are the C_2_H_2_ and H_2_ bonded acids of GaF_3_ with corresponding values of  equal to 287.0 and 287.4 kcal/mol, respectively. Surprisingly, C_2_H_2_/GaF_3_ and H_2_/GaF_3_ outperform HCOOH/GaF_3_-I, CH_3_COOH/GaF_3_-I, H_2_O/GaF_3_, CH_3_OH/GaF_3_ in terms of acidity. Even the ethene bonded to GaF_3_ turns out to be superacidic ( = 301.4 kcal/mol). In general, it can be observed that the BF_3_-bonded acids are weaker than its AlF_3_- or GaF_3_-bonded counterparts. It can also be concluded that the HX/GaF_3_ binary acids tend to be more acidic than corresponding HX/AlF_3_. The values of  indicate that the formation of a triel-bonded binary acid leads to the significant increase of the Brønsted acidity regardless of HX or TF_3_ considered. The boundary values of  correspond to HCl/BF_3_ and H_2_/GaF_3_ and are equal to −31.5 and −108.1 kcal/mol, respectively.

**Table 3:** HX/TF_3_ Interaction Energies Calculated with the Use of Counterpoise Correction (*E*_MP2_), TF_3_ Deformation Energies (*E*_def_), HX/TF_3_ Binding Energies (*E*_b_), SAPT Interaction Energies (*E*_SAPT2+3(CCD)δMP2_), Stabilization Energies (*E*_stab_) Together with Gas Phase Acidities  and Changes in Said Acidities  upon HX/TlF_3_ Formation as Referred to Sole HX Acids^a^^,^^c^

system	*E*_MP2_	*E*_def_	*E*_b_	*E*_SAPT2+3(CCD)δMP2_	*E*_stab_	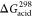	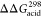
(CH_3_)_2_NH/BF_3_	–57.14	26.83	–30.31	–56.33	–31.77	332.9	–52.0
CH_3_NH_2_/BF_3_	–52.34	25.44	–26.90	–51.91	–35.67	332.5	–59.7
NH_3_/BF_3_	–44.83	23.58	–21.25	–48.13	–38.11	328.9	–63.7
C_2_H_4_/BF_3_	–3.35	0.30	–3.05	–3.30	–1.06	323.1	–75.2
C_2_H_2_/BF_3_	–3.08	0.22	–2.86	–3.25	–0.85	312.1	–56.6
H_2_/BF_3_	–0.67	0.01	–0.66	–0.83	–0.15	325.0	–70.5
H_2_O/BF_3_	–20.50	12.80	–7.70	–20.74	–32.88	304.6	–75.2
CH_3_OH/BF_3_	–30.14	17.12	–13.02	–30.02	–51.28	305.9	–67.2
HCOOH/BF_3_-I	–33.67	21.66	–12.01	–34.85	–55.89	292.4	–41.9
HCOOH/BF_3_-II	–4.43	0.59	–3.84	–4.81	–1.12	289.0	–45.3
CH_3_COOH/BF_3_-I	–39.25	24.17	–15.08	–40.56	–25.01	299.5	–37.2
CH_3_COOH/BF_3_-II	–6.20	1.23	–4.97	–6.62	–1.65	293.3	–43.4
b							
HF/BF_3_	–3.25	0.28	–2.97	–3.61	–1.05	288.1	–74.5
HCl/BF_3_	–2.11	0.11	–2.00	–2.22	–0.70	295.0	–31.5
(CH_3_)_2_NH/AlF_3_	–53.6	7.63	–45.97	–54.64	–14.15	325.1	–59.8
CH_3_NH_2_/AlF_3_	–50.92	7.23	–43.69	–52.24	–17.25	324.81	–67.4
NH_3_/AlF_3_	–46.56	6.77	–39.79	–48.13	–58.71	321.9	–70.8
C_2_H_4_/AlF_3_	–19.85	4.38	–15.47	–20.68	–10.36	312.5	–85.8
C_2_H_2_/AlF_3_	–18.52	4.13	–14.39	–20.05	–11.34	295.3	–73.3
H_2_/AlF_3_	–4.78	0.71	–4.07	–5.37	–2.82	305.0	–90.6
H_2_O/AlF_3_	–34.58	5.15	–29.43	–35.95	–8.29	294.5	–85.3
CH_3_OH/AlF_3_	–39.99	5.94	–34.05	–41.34	–8.67	299.6	–73.5
HCOOH/AlF_3_-I	–49.64	10.25	–39.39	–52.73	–16.11	289.0	–45.3
HCOOH/AlF_3_-II	–27.95	4.51	–23.44	–29.16	–6.60	276.3	–58.1
CH_3_COOH/AlF_3_-I	–53.53	10.85	–42.68	–56.98	–16.14	295.6	–41.2
CH_3_COOH/AlF_3_-II	–32.56	5.26	–27.30	–33.84	–6.67	282.1	–54.6
b							
HF/AlF_3_	–18.64	2.99	–15.65	–19.36	–6.47	266.6	–96.1
F–F interaction							
HCl/AlF_3_	–13.45	2.83	–10.62	–13.92	–8.89	264.9	–61.5
(CH_3_)_2_NH/GaF_3_	–51.47	7.24	–44.23	–50.75	–107.73	321.5	–63.4
CH_3_NH_2_/GaF_3_	–48.18	6.75	–41.43	–48.03	–141.24	320.5	–71.7
NH_3_/GaF_3_	–43.57	6.26	–37.31	–43.96	–114.85	315.7	–77.0
C_2_H_4_/GaF_3_	–20.8	5.20	–15.60	–20.76	–73.17	301.4	–96.8
C_2_H_2_/GaF_3_	–18.37	4.57	–13.80	–19.40	–56.91	287.0	–81.7
H_2_/GaF_3_	–3.13	0.68	–2.45	–3.59	–14.09	287.4	–108.1
H_2_O/GaF_3_	–28.89	3.98	–24.91	–29.17	–51.10	293.9	–86.0
CH_3_OH/GaF_3_	–34.05	4.79	–29.26	–34.08	–63.67	297.7	–75.4
HCOOH/GaF_3_-I	–45.82	9.47	–36.35	–47.71	–78.48	289.1	–45.3
HCOOH/GaF_3_-II	–22.40	3.45	–18.95	–22.64	–42.81	275.1	–59.2
CH_3_COOH/GaF_3_-I	–49.39	9.96	–39.43	–51.54	–63.37	295.5	–41.2
CH_3_COOH/GaF_3_-II	–26.84	4.07	–22.77	–27.06	–27.78	281.4	–55.3
b							
HF/GaF_3_	–14.23	2.08	–12.15	–14.37	–17.41	269.7	–92.9
HCl/GaF_3_	–12.48	2.73	–9.75	–12.41	–26.24	260.6	–65.8

a Rows of data containing the formula of the binary acid describe the triel bonds, which (if those exist) are followed by those characterizing H-bonds.

b There are two H-bond interactions characterized by the same set of numbers in the equilibrium structure of the triel-bonded acid.

c All values are expressed in kcal/mol.

**Figure 4:**
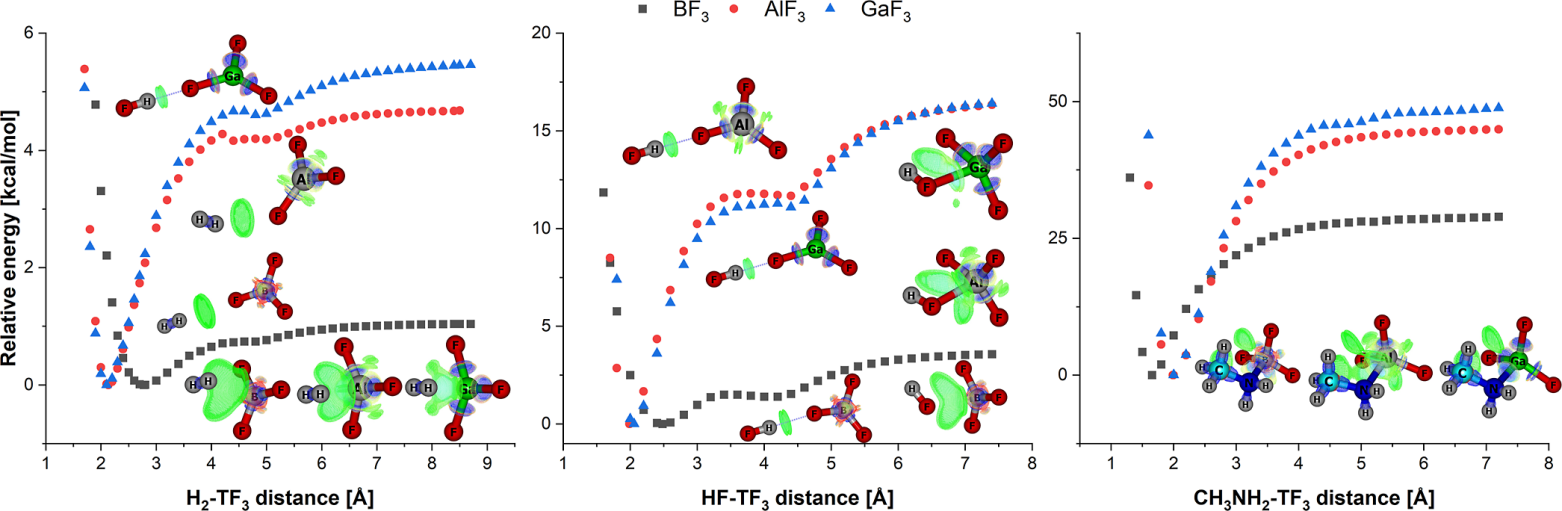
Dissociation curves for H_2_ (left), HF (middle), and CH_3_NH_2_ (right) TF_3_-bonded systems calculated at the MP2/aug-cc-pV(T+d)Z (triel atoms)/aug-cc-pVTZ (remaining atoms) level of theory. Additionally, the structures of minima together with corresponding sgn(λ_2_)ρ(**r**) mapped on DORI with the isovalue equal to 0.9 are also shown. The blue and green colors describe covalent and vdW-type interactions, respectively. The local minima (only H-bonded) graphs are shown near corresponding minima, whereas those describing global minima are shown in available spaces.

The HX/TF_3_ (where HX = H_2_, HF, or CH_3_NH_2_) dissociation curves are depicted on Figure 4. Several things immediately stand out from a glance at the said figure. First, the relative scales of the axes of ordinates iidicate diverse binding energies. Subsequently, the course of the HX/BF_3_ dissociation is different from that of HX/AlF_3_ and HX/GaF_3_. For the last two, the corresponding plots are highly alike. Another factor that stands apart is the presence of two minima along dissociation paths of H_2_- and HF-bonded binary acids. Out of those two minima, the deeper one corresponds to the system held together by both triel as well as the hydrogen bond. However, the shallow minima represent the system bonded by the H-bond only. Thus, the maxima on the presented dissociation curves can be thought of as a point at which triel bonds are broken. This indicates both directionality as well as the partial covalent character of triel interactions.

The results of sgn(λ_2_)ρ(**r**)/DORI analyses confirm the conclusions drawn from the dissociation curves. Moreover, the green color of the lobes corresponding to H_2_-bonded systems confirms their noncovalent character. On the other hand, the bluish lobes between HF and CH_3_NH_2_-bonded compounds legitimize the covalent nature of the interaction. Contrary to the picture visualized in Figure 3, the sgn(λ_2_)ρ(**r**)/DORI of HF/BF_3_ does not indicate the uniqueness of the halogen bonding between HF and BF_3_. It suggests, however, the existence of a vdW interaction between the whole HF moiety and BF_3_ instead. Thus, the HF/BF_3_ binary acid may be regarded as both triel- and H-bonded.

### 3.4 Stoichiometry and Acidity

**Figure 5:**
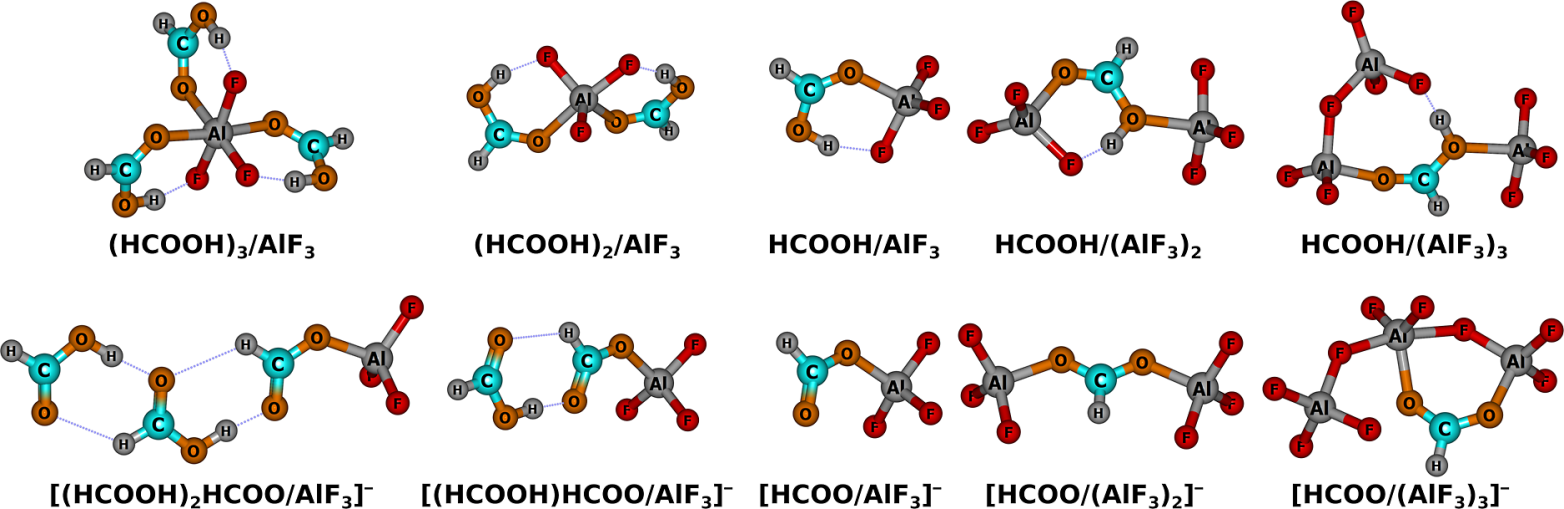
Equilibrium structures of (HCOOH)_*n*_/(AlF_3_)_4–*n*_ (*n* = 1–3) acids and their conjugated bases calculated at the MP2/aug-cc-pV(D+d)Z (Al)/aug-cc-pVDZ (remaining atoms) level.

It is a well-known fact that the acidity of conjugate acids can be controlled by the relative stoichiometry of their constituents. Many examples of such effects are described in Olah’s *Superacid Chemistry*.^12^ It was Gillespie and co-workers^201^ who found that the acidity of the H_2_SO_4_/SO_3_ system can increase hundredfold while changing the percentage concentration of SO_3_ in the mixture from 1 to 45%. On a similar note, Gillespie also found that the acidity of HSO_3_F/SbF_5_ Lewis–Brønsted acid increases 100,000-fold upon a 0 to 50% change in mol % of SbF_5_. It is our research group that studied the effect of the excess of Lewis acid in triel-bonded HClO_4_/AlF_3_ and pnictogen-bonded HClO_4_/SbF_5_ Lewis–Brønsted superacids on the acidity of an entire system. It was found that the aggregation of Lewis acid molecules is preferred over the surrounding of Brønsted acid moiety.^34^ Bearing all of that in mind, we decided to investigate the effect of stoichiometry of the arbitrarily chosen triel-bonded (HCOOH)_*n*_/(AlF_3_)_4–*n*_ (*n* = 1–3) conjugate system on the acidity. The  of mentioned systems are presented in Table 4, whereas the corresponding equilibrium structures of acids and conjugated bases are visualized in Figure 5.

**Table 4:** Gas-Phase Acidities () of (HCOOH)_*n*_/(AlF_3_)_4–*n*_ (*n* = 1–3)^a^

	HCOOH/AlF_3_ molar ratio				
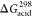	3:1	2:1	1:1	1:2	1:3
	292.5	284.7	289.6	248.8	240.1

a The results were calculated using the MP2 method and aug-cc-pV(D+d)Z (Al)/aug-cc-pVDZ (remaining atoms) basis set.

The results presented in Table 4 indicate that the addition of HCOOH to HCOOH/AlF_3_ binary acid leads to a moderate increase in acidity as the value of  corresponding to HCOOH/(AlF_3_)_2_ is 4.9 kcal/mol smaller than that of HCOOH/AlF_3_. Somewhat surprisingly, addition of a third HCOOH molecule to AlF_3_, and thus formation of HCOOH/(AlF_3_)_3_, leads to increase in acidity as compared to either HCOOH/AlF_3_ or HCOOH/(AlF_3_)_2_. This baffling at first sight phenomenon can be elucidated by the fact that the addition of the second and third HCOOH to AlF_3_ decreases the electron-accepting properties of the latter (vide Figure 5). If, for the sake of discussion, one of the HCOOH molecules attached to AlF_3_ is assumed to be acidic (HCOOH^a^), then a stronger interaction (both triel and H-bond) is formed between HCOOH^a^ and AlF_3_ than between HCOOH^a^ and (HCOOH)_2_/AlF_3_. Altogether, the overall acidity of (HCOOH)_*n*_/AlF_3_ with *n* = 1–3 is a result of the balance of two factors: (i) decrease of the acidity as an effect of the decreased electron-accepting properties of HCOOH-bonded AlF_3_ and (ii) increase of the acidity upon the stabilization of the corresponding Brønsted base.^66^

As can be concluded from the analysis of the results presented in Table 4, decreasing the HCOOH/AlF_3_ molar ratio, i.e., solvating HCOOH with up to three AlF_3_ molecules, leads to a significant increase in acidity. The surge in acidity upon addition of consecutive AlF_3_ molecules in HCOOH/(AlF_3_)_*n*_ is expected to decrease with the number of *n*. For example, the value of  decreases by 40 kcal/mol upon addition of the second AlF_3_. However, further solvation of formic acid leads to a less spectacular increase of acidity as the Gibbs free energy of deprotonation of HCOOH/(AlF_3_)_3_ is 8.7 kcal/mol lower than that of HCOOH/(AlF_3_)_2_. With  of HCOOH/(AlF_3_)_3_ equal to 240.1 kcal/mol, its acidity is as high as that of H(CHAl_11_H_10_Cl) caralumane acid.^65^ The change in the acidity is expected to decrease upon further reduction of the HCOOH/AlF_3_ ratio. The trends observed for HCOOH/(AlF_3_)_*n*=1–3_ concur with those observed for HClO_4_/(AlF_3_)_*n*=1–3_.^34^

Structure-wise, addition of AlF_3_ to HCOOH/AlF_3_ leads to the formation of a second triel bond between HCOOH and two AlF_3_ molecules. Altogether, the whole molecule is held in tandem by two triel and one H-bond interactions. Analogously, Brønsted base conjugated to the discussed acid, i.e., [HCOO/(AlF_3_)_2_]^−^, is also kept by two triel interactions. Contrary to that, addition of yet another Lewis acid molecule leads to the formation of a triel interaction between two AlF_3_ moieties. Altogether, in HCOOH/(AlF_3_)_3_ acid, two AlF_3_ subsystems are connected to the oxygen atoms of Brønsted acid via triel bonds, whereas the remaining AlF_3_ interacts in a H-bond mode with the acidic hydrogen of formic acid. This factor explains the only 8.7 kcal/mol increase in the acidity upon the addition of a third AlF_3_ to HCOOH. Namely, for a studied example, the effect of the formation of a H-bond by a Brønsted acid leads only to a fraction of the acidity increase caused by creation of the triel bond. When it comes to structure of (HCOOH)_*n*_/AlF_3_ vs addition of up to three consecutive HCOOH molecules to AlF_3_, it leads to the formation of a pair of triel and H-bond between HCOOH and AlF_3_. When it comes to their corresponding bases, another trend is observed. Namely, upon the addition of HCOOH to [HCOOH_*n*_(HCOO)/AlF_3_]^−^ (*n* = 0 or 1), the formation of two H-bonds with formic acid entities already being part of the conjugate acid is preferred over the interaction with AlF_3_ upon the addition. As an effect, the excess electron charge is smeared over AlF_3_ and H-bonded chain of HCOOH moieties. Further increase of the HCOOH/AlF_3_ ratio is expected to induce the formation of a H-bonded formic acid chain and as such, it is not expected to increase acidity substantially.

## 4 Conclusions

In summary, this study delved into both the nature of triel interaction as well as the effects of its formation on Brønsted acidity of HX/TF_3_ binary systems. Through a combination of various theoretical methods, several key findings have emerged:

i depending on the structure of HX or to be more specific, the number of electron density donor atoms in HX, a diverse number of HX/TF_3_ isomers may exist. The higher the nucleophilic properties of a triel bond acceptor atom, the stronger the interaction. For the studied systems, the importance of nonglobal minimum isomers is inversely proportional to the atomic number of a triel atom,
ii the presence of halogen atoms in either HX or TF_3_ may cause the formation of halogen bonds, which were found to diminish the strength of triel bonds,
iii the values of *H*_BCP_/ρ_BCP_ calculated for AlF_3_-bonded systems suggest that the Al-based triel interactions are characterized by the highest closed-shell character out of all TF_3_ compounds considered,
iv the HX/TF_3_ interaction energies obtained with either counterpoise correction (*E*_MP2_) or SAPT (*E*_SAPT2+3(CCD)δMP2_) do not differ significantly. The interaction energies were wound to span the −57.41 to −0.67 kcal/mol range depending on the system considered and applied method of determination,
v the interaction between HX and TF_3_ is dominated by electrostatics in all but two binary acids, i.e., H_2_/BF_3_ and H_2_/AlF_3_, for which binding is dominated by dispersion and induction contributions, respectively,
vi most of the studied binary acids turned out to be superacidic. Even as weak Brønsted acids such as H_2_, C_2_H_2_, or C_2_H_4_ turned out to be superacidic upon triel-bonding with GaF_3_,
vii formation of the triel bond may cause a significant increase in acidity, as the absolute values of  may approach or even exceed (for H_2_/GaF_3_) 100 kcal/mol,
viii the dissociation curves of HX/BF_3_ differ significantly from those obtained for HX/AlF_3_ and HX/GaF_3_ in terms of depth of the observed minima. For the HX/TF_3_ systems held together by more than one type of interaction, triel is always a stronger one,
ix the sgn(λ_2_)ρ(**r**)/DORI analysis reveals a partially covalent character of the interaction between some of HX and TF_3_,
x the solvation of HCOOH with AlF_3_ is expected to increase the acidity of the resulting acids. The reverse process, i.e., solvation of AlF_3_ with HCOOH, is expected to lead to the increase in acidity only to a 1:2 AlF_3_ to HCOOH ratio. Exceeding this ratio induces decrease in acidity.

The nature and strength of triel interactions is highly dependent on the type of systems connected. Beyond a shadow of doubt, formation of triel bond by Brønsted acids leads to increase in acidity. Due to the significant strength of triel interactions, various properties of both donor and acceptor moieties are expected to change upon triel bonding.

## Supporting Information Available

Cartesian coordinates of all studied systems together with corresponding graphics, IBSI/NBO plot,  values of HA systems, and contributions to total *E*_SAPT2+3(CCD)δMP2_ energies (PDF)
