# Supplementary material for: Can H2 be Superacidic? A Computational Study of Triel-Bonded Brønsted Acids
Source: J Phys Chem A. 2024 Jun 13;128(25):5009–20. doi: 10.1021/acs.jpca.4c02663 (PMC11215784; doi:10.1021/acs.jpca.4c02663)
Supplement: Supplementary file 1 — jp4c02663_si_001.pdf [file jp4c02663_si_001.pdf]

Supporting Information

Can H_2 be Superacidic? A Computational Study of Triel-Bonded Brønsted Acids

Jakub Brzeski [a*]

[a] *Faculty of Chemistry, University of Gdańsk,*

Wita Stwosza 63, 80-308 Gdańsk, Poland

[*] corresponding author: jakub.brzeski@ug.edu.pl

Table S1. Cartesian coordinates (in Å) of the equilibrium structures of HX/TF_3.

System	Coordinates			
$(CH_3)_2NH/BF_3$ 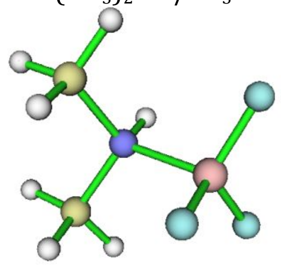	B	-0.173299059747	-0.764092502684	0.000000000000
	F	-1.522957062237	-0.481899462411	0.000000000000
	F	0.262935939392	-1.384608956967	1.152289582278
	F	0.262935939392	-1.384608956967	-1.152289582278
	N	0.578963731574	0.706101735418	0.000000000000
	C	0.262935939392	1.476222043227	-1.221324590945
	H	0.796130369743	2.425159551013	-1.216591582004
	H	-0.808748188487	1.652907590242	-1.234451097195
	H	0.541541745303	0.887286262735	-2.089027152010
	H	1.574436700337	0.492435481453	0.000000000000
	C	0.262935939392	1.476222043227	1.221324590945
	H	0.796130369743	2.425159551013	1.216591582004
	H	-0.808748188487	1.652907590242	1.234451097195
	H	0.541541745303	0.887286262735	2.089027152010
CH_3NH_2/BF_3 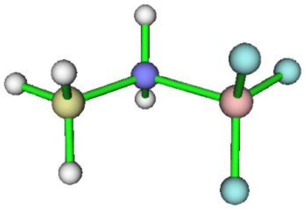	B	0.536143364765	0.237420294112	0.000000000000
	F	0.883399170086	-0.429606470942	1.154099664809
	F	0.883399170086	1.570494303373	0.000000000000
	F	0.883399170086	-0.429606470942	-1.154099664809
	N	-1.117298863836	0.250096318650	0.000000000000
	C	-1.721165047027	-1.100434905678	0.000000000000
	H	-2.806204265618	-1.043367293415	0.000000000000
	H	-1.372250678497	-1.623687978334	0.883863258533
	H	-1.372250678497	-1.623687978334	-0.883863258533
	H	-1.416853245487	0.777022356085	0.815949491788
	H	-1.416853245487	0.777022356085	-0.815949491788
NH_3/BF_3 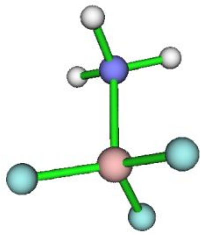	B	0.000000000000	0.000000000000	-0.207519123603
	F	0.000000000000	1.332603962278	-0.542319323645
	F	-1.154068882569	-0.666301981139	-0.542319323645
	F	1.154068882569	-0.666301981139	-0.542319323645
	N	0.000000000000	0.000000000000	1.462773866003
	H	-0.001408354934	-0.952032697266	1.813600114891
	H	-0.823780320220	0.477236019111	1.813600114891
	H	0.825188677842	0.474796676567	1.813600114891
C_2H_4/BF_3 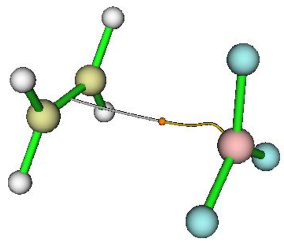	B	-0.872072903670	-0.002854382330	0.000000000000
	F	-0.905994414189	1.316624481558	0.000000000000
	F	-0.905994414189	-0.662626416511	1.141045041997
	F	-0.905994414189	-0.662626416511	-1.141045041997
	C	2.050791979061	-0.599878054729	0.000000000000
	C	2.066515168655	0.613024373376	0.000000000000
	H	2.079609093462	1.675496658847	0.000000000000
	H	2.038761684649	-1.662447454809	0.000000000000
C_2H_2/BF_3 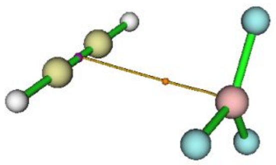	B	-0.872072903670	-0.002854382330	0.000000000000
	F	-0.905994414189	1.316624481558	0.000000000000
	F	-0.905994414189	-0.662626416511	1.141045041997
	F	-0.905994414189	-0.662626416511	-1.141045041997
	C	2.050791979061	-0.599878054729	0.000000000000
	C	2.066515168655	0.613024373376	0.000000000000
	H	2.079609093462	1.675496658847	0.000000000000

	H	2.038761684649	-1.662447454809	0.000000000000
H_2/BF_3 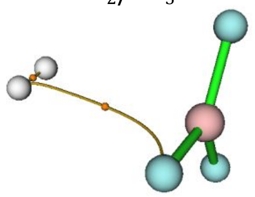	B	0.156598332662	0.005011438964	0.000000000000
	F	0.176717196048	-1.312335806878	0.000000000000
	F	0.156598332662	0.663819160863	1.140586605162
	F	0.156598332662	0.663819160863	-1.140586605162
	H	-2.611259087203	0.287701181169	0.000000000000
	H	-2.580957323695	-0.450480985151	0.000000000000
H_2O/BF_3 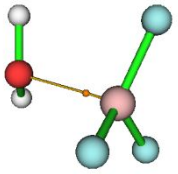	B	-0.277334994686	0.039059321164	0.000000000000
	F	-0.527475128153	-0.630038432659	1.152415584673
	F	-0.527475128153	-0.630038432659	-1.152415584673
	F	-0.527475128153	1.357832387080	0.000000000000
	H	1.796503773837	-0.505841746781	-0.775139326470
	O	1.504436985670	-0.007926596020	0.000000000000
	H	1.796503773837	-0.505841746781	0.775139326470
CH_3OH/BF_3 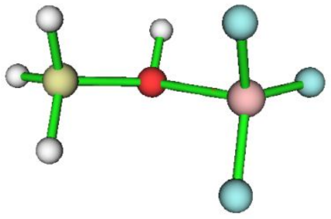	B	0.621766175150	-0.078168449761	0.016522685823
	F	0.491971491433	-0.417581624410	1.333598974259
	F	1.507000119177	0.921007934758	-0.234602835903
	F	0.611894209525	-1.123998570606	-0.846382634131
	H	-0.838572067896	1.548634975487	0.104749546334
	O	-0.836112843634	0.689340760445	-0.338396384378
	C	-2.029302927079	-0.059127739511	0.005831091368
	H	-2.059889456739	-0.239193534794	1.076001379189
	H	-2.894566018068	0.501663036191	-0.331371039920
	H	-1.948875409588	-0.995071519027	-0.533330295551
$HCOOH/BF_3$ – I 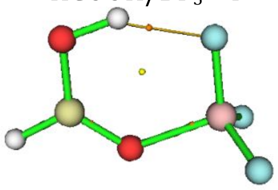	B	0.062650966180	-0.868532591455	0.000000000000
	F	0.442313849231	-1.452441008161	1.152155658100
	F	0.442313849231	-1.452441008161	-1.152155658100
	F	-1.282558182852	-0.465082587270	0.000000000000
	H	-1.327104900476	1.167147307774	0.000000000000
	O	-0.817731432673	2.017403635787	0.000000000000
	O	0.921923538402	0.568240282713	0.000000000000
	C	0.442313849231	1.708851542243	0.000000000000
	H	1.107804513804	2.566936423226	0.000000000000
$HCOOH/BF_3$ – II 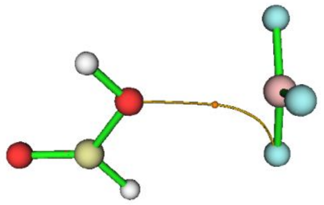	B	-1.420856619546	-0.089537050357	0.004781209294
	F	-1.065027857035	-1.363894382348	-0.037515996249
	F	-1.697845544482	0.551761602635	-1.114545882699
	F	-1.654346656020	0.487263209357	1.168434637267
	H	1.255431583382	1.459631346910	-0.005487375981
	O	0.905736826579	0.553851672849	-0.024747667854
	O	3.115825371290	0.068004493655	0.016603900031
	C	1.969332544767	-0.290116982205	-0.011648302840
	H	1.615339127709	-1.322267389612	-0.030733543726
CH_3COOH/BF_3 – I 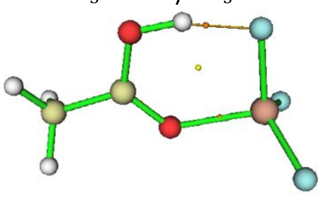	B	-1.227120796356	-0.098224047360	0.009346802344
	F	-1.609307126208	-0.354734575833	1.282336949720
	F	-1.880574893650	-0.752677969503	-0.971600179654
	F	-1.093568576868	1.281270011967	-0.244178553816
	H	0.479336615189	1.597248367352	-0.051035071583
	O	1.410861551417	1.263555015082	0.005447669854
	O	0.300407213761	-0.694346390921	-0.067538481648
	C	1.359546609153	-0.039324389858	-0.023957030900
	C	2.675801026477	-0.726674554036	0.001233960312
	H	3.286144905416	-0.357978647745	-0.820305345520
	H	3.182563374980	-0.472256005576	0.930642485010

	H	2.536378553805	-1.798286005769	-0.071995149467
CH_3COOH/BF_3 – II 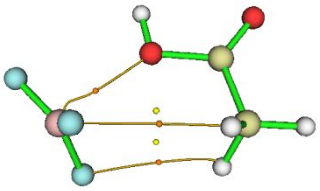	B	-0.359701059893	-1.556135930697	0.000000000000
	F	-0.976089290801	-1.294371779180	1.141305386620
	F	0.746335605545	-2.281757892975	0.000000000000
	F	-0.976089290801	-1.294371779180	-1.141305386620
	H	1.831070616011	0.359976519406	0.000000000000
	O	0.861115092789	0.394559298812	0.000000000000
	O	1.343128170613	2.591230892518	0.000000000000
	C	0.508891455064	1.721481039929	0.000000000000
	C	-0.976089290801	1.884863033862	0.000000000000
	H	-1.394921938912	1.395197481505	-0.876801351103
	H	-1.394921938912	1.395197481505	0.876801351103
	H	-1.220893718954	2.940435297807	0.000000000000
HF/BF_3 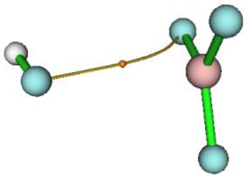	B	-0.571835438858	0.059928725479	0.000000000000
	F	-0.608785520733	0.713779224158	1.142565362941
	F	-0.608785520733	-1.263331755201	0.000000000000
	F	-0.608785520733	0.713779224158	-1.142565362941
	H	2.149537866627	-0.980059384902	0.000000000000
	F	1.905205385204	-0.088624942458	0.000000000000
HCl/BF_3 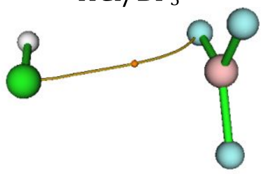	B	1.085698144810	-0.108788755747	0.000000000000
	F	1.108886284395	-0.764586918279	1.141747921678
	F	1.108886284395	1.212269971332	0.000000000000
	F	1.108886284395	-0.764586918279	-1.141747921678
	H	-1.830782558376	1.386758342658	0.000000000000
	Cl	-1.972802227694	0.118195306806	0.000000000000
$(CH_3)_2NH/AlF_3$ 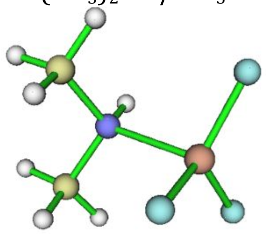	Al	-0.211786034811	-0.805204833503	0.000000000000
	F	-1.809900472699	-0.321041981042	0.000000000000
	F	0.362468225626	-1.467120305672	1.419869391762
	F	0.362468225626	-1.467120305672	-1.419869391762
	N	0.692153956661	0.962236864492	0.000000000000
	C	0.362468225626	1.730877412793	-1.222046409880
	H	0.871025540183	2.694508638170	-1.218754858598
	H	-0.713262059233	1.887003883924	-1.243400024237
	H	0.656485044656	1.154329587032	-2.094788558725
	H	1.694701157393	0.787334962313	0.000000000000
	C	0.362468225626	1.730877412793	1.222046409880
	H	0.871025540183	2.694508638170	1.218754858598
	H	-0.713262059233	1.887003883924	1.243400024237
	H	0.656485044656	1.154329587032	2.094788558725
CH_3NH_2/AlF_3 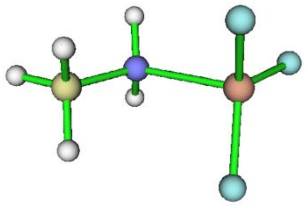	Al	0.575261771585	0.227387968730	0.000000000000
	F	0.884908129595	-0.598532129439	1.416126145364
	F	0.884908129595	1.864523624745	0.000000000000
	F	0.884908129595	-0.598532129439	-1.416126145364
	N	-1.412105816466	0.193498853384	0.000000000000
	C	-1.958369320402	-1.188613583967	0.000000000000
	H	-3.045165158747	-1.186867050332	0.000000000000
	H	-1.588247834945	-1.699881675658	0.883207941303
	H	-1.588247834945	-1.699881675658	-0.883207941303
	H	-1.757152507860	0.700321019025	0.810578295085
	H	-1.757152507860	0.700321019025	-0.810578295085
NH_3/AlF_3	Al	0.259819493391	0.000760429628	-0.000690562456
	F	0.568745710487	-1.620878560443	0.221850216663
	F	0.560973329682	1.007856367278	1.291537722797

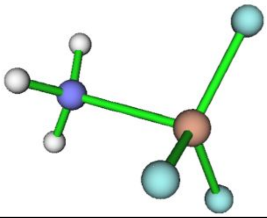	F	0.551164992330	0.619939481745	-1.519527776701
	N	-1.740243855393	-0.006933239940	0.006171799441
	H	-2.110169465891	-0.026331113188	-0.938919711494
	H	-2.109106391132	0.820632863903	0.463854721424
	H	-2.104626900427	-0.817910273416	0.496098245812
C_2H_4/AlF_3 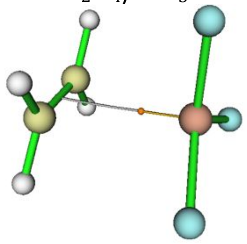	Al	-0.560658649139	0.000555336766	0.015405203599
	F	-0.877389452238	1.418157058852	-0.779093799259
	F	-0.657084041551	-0.000827613421	1.669079797316
	F	-0.882957598130	-1.414156081967	-0.781942222804
	C	1.812048434903	-0.674441589788	-0.074201976290
	C	1.815041180959	0.669967597235	-0.074046744261
	H	1.754386953472	1.227198341990	0.852190793656
	H	1.749236423682	-1.231518814932	0.851963448527
	H	1.887029321474	-1.237188213598	-0.993792222444
	H	1.892251904039	1.232572983637	-0.993531316893
C_2H_2/AlF_3 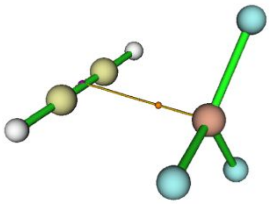	Al	0.492586095352	0.005428716048	0.000000000000
	F	0.731322508317	-1.634649017196	0.000000000000
	F	0.731322508317	0.836437102285	1.409641792567
	F	0.731322508317	0.836437102285	-1.409641792567
	C	-1.857020103025	0.578387335839	0.000000000000
	C	-1.876377290375	-0.637679005995	0.000000000000
	H	-1.884567636538	-1.703304399396	0.000000000000
	H	-1.864374915354	1.644454401739	0.000000000000
H_2/AlF_3 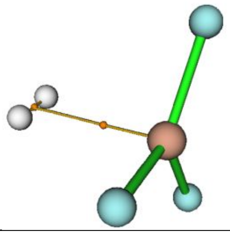	Al	0.036124612093	0.002102211386	0.000000000000
	F	0.135764786297	-1.640134711981	0.000000000000
	F	0.135764786297	0.827253919059	1.418299481617
	F	0.135764786297	0.827253919059	-1.418299481617
	H	-2.086529234307	0.295077020356	0.000000000000
	H	-2.048739951400	-0.451763855268	0.000000000000
H_2O/AlF_3 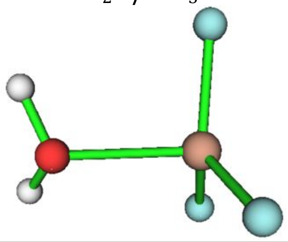	Al	-0.258185127341	0.057103122173	0.000000000000
	F	-0.521029668296	-0.755901008206	1.428055134083
	F	-0.521029668296	-0.755901008206	-1.428055134083
	F	-0.521029668296	1.689629385052	0.000000000000
	H	2.007917159912	-0.605835045355	-0.779274677334
	O	1.676046675096	-0.141389608660	0.000000000000
	H	2.007917159912	-0.605835045355	0.779274677334
CH_3OH/AlF_3 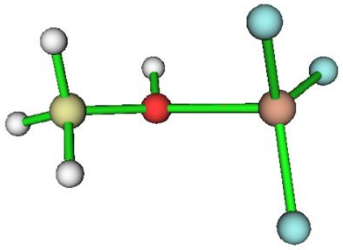	Al	0.619277930807	-0.082134194554	0.037631971476
	F	0.305643611027	-0.584871270586	1.594263670081
	F	1.508974236071	1.311540802730	-0.140584628462
	F	0.776032482414	-1.299759163416	-1.077852721670
	H	-1.146211050222	1.613224360793	-0.256939109656
	O	-1.081235974077	0.665602646946	-0.426294792403
	C	-2.303837268637	-0.020728995863	-0.048156349942
	H	-2.426385670574	0.010978123032	1.029835601437
	H	-3.137813548545	0.445262398403	-0.561888118392
	H	-2.183144363222	-1.044360860743	-0.383364476849
$HCOOH/AlF_3$ – I	Al	-0.063094895620	0.838278231645	0.000000000000
	F	-0.476201909927	1.556159971220	1.429523897288
	F	-0.476201909927	1.556159971220	-1.429523897288

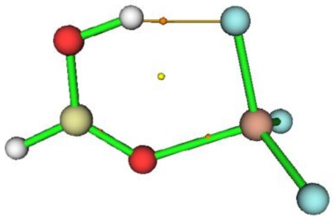	F	1.512204027076	0.165735105985	0.000000000000
	H	1.295892904474	-1.366445466489	0.000000000000
	O	0.783657344478	-2.234607723830	0.000000000000
	O	-0.975197018582	-0.821304045867	0.000000000000
	C	-0.476201909927	-1.959237266343	0.000000000000
	H	-1.124332280363	-2.830949197044	0.000000000000
$HCOOH/AlF_3$ – II 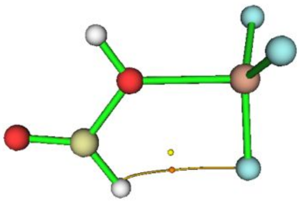	Al	-1.007695439185	-0.071079498727	-0.001993975313
	F	-0.575327363105	-1.677962021565	-0.046648906790
	F	-1.565965678561	0.629099719846	-1.393345689223
	F	-1.564818422286	0.548630630190	1.427380284921
	H	1.105230294034	1.500021594206	0.026929721509
	O	0.850530923379	0.562202052795	0.012217617072
	O	3.094224197232	0.261386896294	0.005640899720
	C	2.012543550221	-0.230706187371	-0.001784146458
	H	1.716511324713	-1.278377539707	-0.019642519616
CH_3COOH/AlF_3 – I 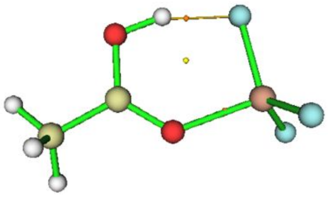	Al	1.198969185847	-0.071727975812	0.001506594431
	F	1.878026381311	-0.557418951936	-1.426408535571
	F	1.839603837281	-0.616065888407	1.426295773191
	F	0.785432183804	1.589995014176	0.031940058858
	H	-0.769264009060	1.578252406976	0.021084653141
	O	-1.704800118891	1.211348980664	0.005725286875
	O	-0.563377217879	-0.721238316157	-0.033053582465
	C	-1.639365044839	-0.084735012890	-0.019850952692
	C	-2.938349979605	-0.805212310778	-0.007135036075
	H	-2.871977796147	-1.677336777478	-0.650192682657
	H	-3.115003109221	-1.150042383464	1.011882316038
	H	-3.746207303485	-0.148202466756	-0.308263386079
CH_3COOH/AlF_3 – II 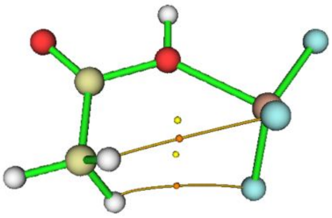	Al	0.831852300016	-0.878057125757	0.000000000000
	F	0.156833714936	-1.466222440050	1.398696983570
	F	2.436219032719	-0.455756670652	0.000000000000
	F	0.156833714936	-1.466222440050	-1.398696983570
	H	0.791126263297	1.678158970754	0.000000000000
	O	0.156833714936	0.941686906332	0.000000000000
	O	-1.298503653350	2.657099124607	0.000000000000
	C	-1.164083588465	1.472005289027	0.000000000000
	C	-2.171191339272	0.380391822065	0.000000000000
	H	-2.023211546598	-0.245638063299	-0.879648456997
	H	-2.023211546598	-0.245638063299	0.879648456997
	H	-3.162752185302	0.817002745018	0.000000000000
HF/AlF_3 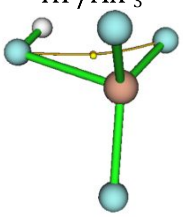	Al	-0.047711167675	0.297864650432	0.000000000000
	F	0.565643265987	0.852659343068	1.423620369439
	F	-1.600495155530	-0.311944543052	0.000000000000
	F	0.565643265987	0.852659343068	-1.423620369439
	H	-0.247666610307	-2.062024073093	0.000000000000
	F	0.565643265987	-1.594509292993	0.000000000000
HCl/AlF_3	Al	0.673270097194	-0.111406692966	0.000000000000
	F	0.870203415701	-0.927696899231	1.418517751357
	F	0.870203415701	1.536321163781	0.000000000000
	F	0.870203415701	-0.927696899231	-1.418517751357
	H	-1.618567056010	1.441064104774	0.000000000000
	Cl	-1.801731558214	0.169345687118	0.000000000000

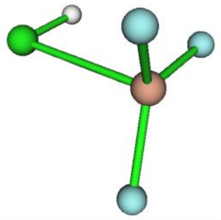				
$(CH_3)_2NH/GaF_3$ 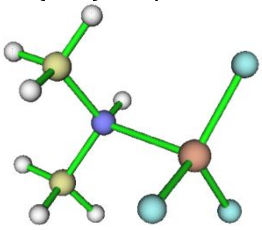	Ga	-0.168567192770	-0.627565301619	0.000000000000
	F	-1.851829509225	-0.107027499872	0.000000000000
	F	0.427856674929	-1.332418633822	1.498764269062
	F	0.427856674929	-1.332418633822	-1.498764269062
	N	0.753268298475	1.144237303751	0.000000000000
	C	0.427856674929	1.908953780496	-1.223949728150
	H	0.950184942607	2.864884016371	-1.225460851994
	H	-0.646199087794	2.077360915670	-1.240838298342
	H	0.711512879712	1.322234564747	-2.093540356597
	H	1.752472750026	0.955241823899	0.000000000000
	C	0.427856674929	1.908953780496	1.223949728150
	H	0.950184942607	2.864884016371	1.225460851994
	H	-0.646199087794	2.077360915670	1.240838298342
	H	0.711512879712	1.322234564747	2.093540356597
CH_3NH_2/GaF_3 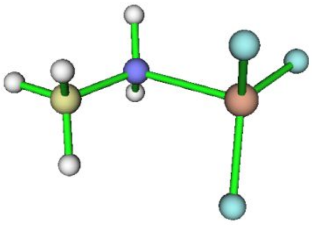	Ga	0.434131064481	0.177686637126	0.000000000000
	F	0.761833915976	-0.697054196196	1.491465406643
	F	0.761833915976	1.903896724575	0.000000000000
	F	0.761833915976	-0.697054196196	-1.491465406643
	N	-1.567546913099	0.132563051741	0.000000000000
	C	-2.099506549615	-1.253762695925	0.000000000000
	H	-3.186024813326	-1.258132583438	0.000000000000
	H	-1.723777732691	-1.760128971133	0.883645062878
	H	-1.723777732691	-1.760128971133	-0.883645062878
	H	-1.912065400930	0.638322300318	0.810575649199
	H	-1.912065400930	0.638322300318	-0.810575649199
NH_3/GaF_3 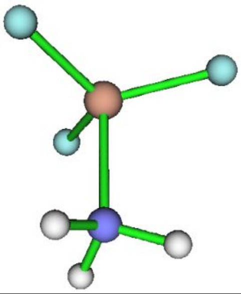	Ga	0.000000000000	0.000000000000	0.186399230433
	F	0.000000000000	1.728220186280	0.502959074884
	F	1.496682581016	-0.864110093140	0.502959074884
	F	-1.496682581016	-0.864110093140	0.502959074884
	N	0.000000000000	0.000000000000	-1.827831754908
	H	-0.821557114303	-0.474326221588	-2.187816299817
	H	0.821557114303	-0.474326221588	-2.187816299817
	H	0.000000000000	0.948652445293	-2.187816299817
C_2H_4/GaF_3 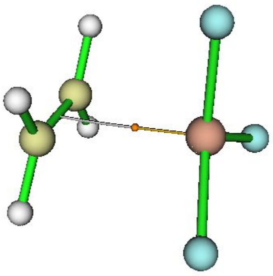	Ga	-0.388281747425	-0.000386020960	0.017438702912
	F	-0.788863273834	1.493840126590	-0.799074553203
	F	-0.467713627447	-0.009511375622	1.765942226634
	F	-0.786706389701	-1.486001815592	-0.815580268946
	C	1.900494686376	-0.672039945302	-0.119309838232
	C	1.899143913822	0.675377328625	-0.120914493626
	H	1.855341785629	1.228727246190	0.808518128678
	H	1.857728830115	-1.223088015918	0.811526226166
	H	1.958894407111	-1.235866820965	-1.038997829244
	H	1.956487132180	1.237227483842	-1.041886957104
C_2H_2/GaF_3	Ga	-0.110644742817	-0.326666107537	0.000000000000
	F	0.623382541765	-0.835355516906	1.502787397307

Structure	Atom	x	y	z
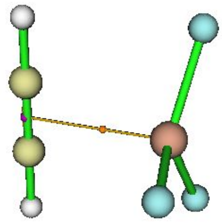	F	0.623382541765	-0.835355516906	-1.502787397307
	F	-1.838779537881	-0.099194150439	0.000000000000
	C	0.623382541765	1.860359121506	-0.608637303477
	C	0.623382541765	1.860359121506	0.608637303477
	H	0.638763279595	1.865743267177	1.674780242812
	H	0.638763279595	1.865743267177	-1.674780242812
H_2/GaF_3 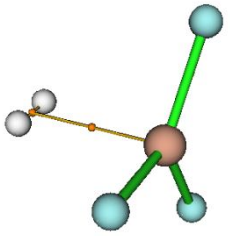	Ga	0.021056096037	0.002254183504	0.000000000000
	F	0.127742822747	-1.728108476963	0.000000000000
	F	0.127742822747	0.871617387894	1.493961122968
	F	0.127742822747	0.871617387894	-1.493961122968
	H	-2.074722898291	0.270238629350	0.000000000000
	H	-2.027072291513	-0.476255024506	0.000000000000
H_2O/GaF_3 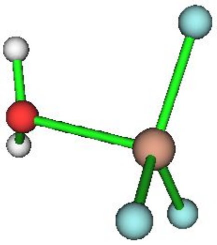	Ga	-0.199720910482	0.051230082184	0.000000000000
	F	-0.453250227375	-0.813635346761	1.503090520619
	F	-0.453250227375	-0.813635346761	-1.503090520619
	F	-0.453250227375	1.772215670547	0.000000000000
	H	2.070024132410	-0.688566267207	-0.777811115941
	O	1.786132010884	-0.189438097462	0.000000000000
	H	2.070024132410	-0.688566267207	0.777811115941
CH_3OH/GaF_3 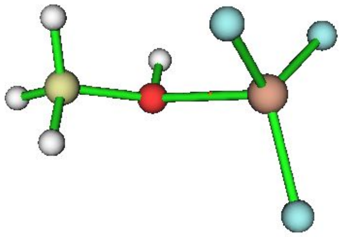	Ga	0.486385875283	-0.050852641431	0.051069982306
	F	-0.035347390047	-0.844234899473	1.525998048092
	F	1.164429913807	1.560510353259	0.195248531919
	F	1.055893792042	-1.095836059045	-1.223127439623
	H	-1.332268379234	1.536411795968	-0.465681440758
	O	-1.272719931110	0.579351253365	-0.584280888697
	C	-2.488418383628	-0.074561356436	-0.134462916600
	H	-2.592863891174	0.032412784642	0.940335276511
	H	-3.329414347914	0.356761931134	-0.666655191077
	H	-2.375932583420	-1.120551065497	-0.393215728360
$HCOOH/GaF_3$ – I 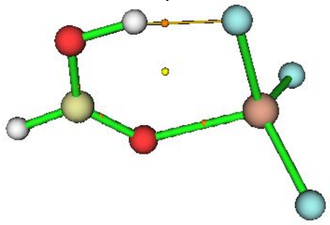	Ga	-0.057223428504	0.664031916576	0.000000000000
	F	-0.466021948855	1.414142021728	1.513887990793
	F	-0.466021948855	1.414142021728	-1.513887990793
	F	1.592210287727	-0.075827378607	0.000000000000
	H	1.305565857119	-1.544015067599	0.000000000000
	O	0.795803586916	-2.423622650945	0.000000000000
	O	-0.992777683822	-1.043319545752	0.000000000000
	C	-0.466021948855	-2.170870250393	0.000000000000
	H	-1.101212637481	-3.052325260308	0.000000000000
$HCOOH/GaF_3$ – II 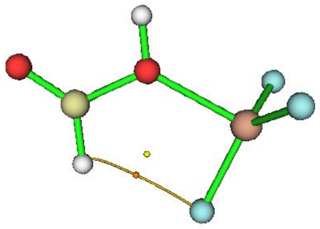	Ga	-0.803571850722	-0.057818251778	0.000445033105
	F	-0.311794126535	-1.740243363258	-0.017697030946
	F	-1.384574639823	0.651672383956	-1.480009163711
	F	-1.364749532782	0.622118226190	1.502363579248
	H	1.369386056836	1.530641202736	-0.000416916101
	O	1.104815879455	0.595373375188	-0.006705987893
	O	3.344781655225	0.273671067195	0.003743150606
	C	2.257265056299	-0.206767008742	-0.003674927102
	H	1.951035272988	-1.251954068631	-0.009543304854
CH_3COOH/GaF_3 – I	Ga	-0.971694918257	-0.054227485688	0.000683168665
	F	-1.659654044531	-0.606617497419	1.501082890929

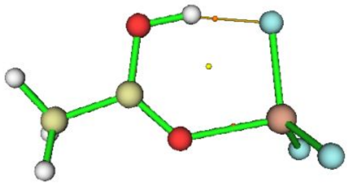	F	-1.685914280300	-0.518706037237	-1.517008072771
	F	-0.479885296324	1.684590310574	0.047674341965
	H	1.023400806090	1.571288751886	0.030071743969
	O	1.962916196538	1.194587899856	0.009836981029
	O	0.831750142348	-0.747786419058	-0.034110832941
	C	1.902688353619	-0.098880776435	-0.020236745449
	C	3.203640203177	-0.816873107175	-0.010724933203
	H	3.395025807326	-1.139773714823	1.012794718159
	H	3.132644651012	-1.701230135707	-0.635926646119
	H	4.005251796157	-0.162522348137	-0.333899580052
CH_3COOH/GaF_3 – II 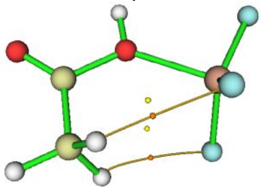	Ga	0.125073572268	0.974238006529	0.000000000000
	F	1.047690772083	0.788462459111	1.473940955903
	F	-1.297041515444	1.987208332158	0.000000000000
	F	1.047690772083	0.788462459111	-1.473940955903
	H	-1.857032051847	-0.731014955792	0.000000000000
	O	-0.885153117494	-0.744027673058	0.000000000000
	O	-1.250637205756	-2.964090573900	0.000000000000
	C	-0.436614194855	-2.092311442980	0.000000000000
	C	1.047690772083	-2.147897803455	0.000000000000
	H	1.426234637344	-1.628309600755	-0.880341404021
	H	1.426234637344	-1.628309600755	0.880341404021
	H	1.362084945414	-3.184741849545	0.000000000000
HF/GaF_3 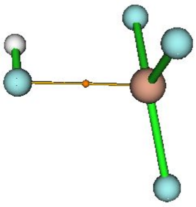	Ga	-0.035955426401	0.239101245223	0.000000000000
	F	0.593787647935	0.818691185574	1.505275011929
	F	-1.630565382824	-0.500659044072	0.000000000000
	F	0.593787647935	0.818691185574	-1.505275011929
	H	-0.242559856704	-2.148346762725	0.000000000000
	F	0.593787647935	-1.721589082048	0.000000000000
HCl/GaF_3 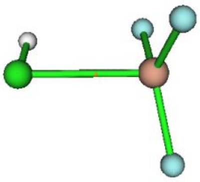	Ga	0.502677606034	-0.111053930183	0.000000000000
	F	0.713914047938	-0.963362779050	1.497944949219
	F	0.713914047938	1.628795766478	0.000000000000
	F	0.713914047938	-0.963362779050	-1.497944949219
	H	-1.647783244142	1.521094247194	0.000000000000
	Cl	-1.953582462385	0.270761514681	0.000000000000

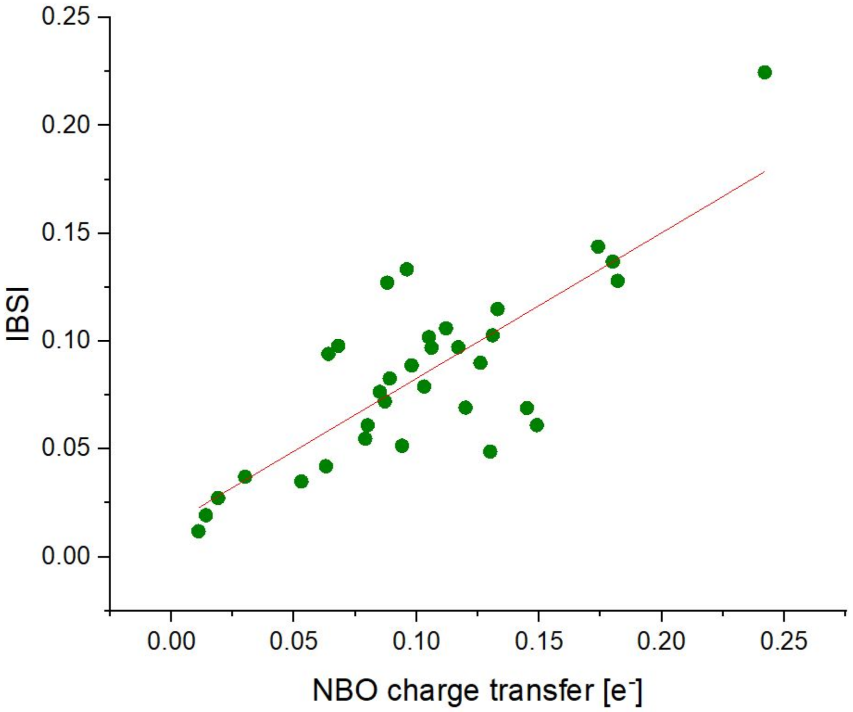

Figure S1. The IBSI/NBO charge transfer dependence. The corresponding R^2 is equal to 0.631 whereas the equation of the regression line is given by $IBSI = 0.675\ NBO + 0.015$.

Table S2. Gibbs free energies of deprotonation ΔG_{acid}^{298} (in kcal/mol, obtained at T=298.15K) of HA used as HA/PnX_3 building blocks.

**Acid**	ΔG_{acid}^{298} $[kcal/mol]$
HBr	318.3
HCl	326.5
H_2O	379.8
NH_3	392.6
CH_3NH_2	392.2
$(CH_3)_2NH$	384.9

Table S3. Contributions to total $E_{SAPT2+3(CCD)\delta_{MP2}}$ interaction energies (in kcal/mol) together with percentage contribution of each attractive interaction type to the total attractive interaction.

System	Exchange	Electrostatics	Induction	Dispersion	%Electrostatics	%Induction	%Dispersion
$(CH_3)_2NH/BF_3$	141.78	-107.67	-62.61	-27.83	54.35	31.60	14.05
CH_3NH_2/BF_3	137.29	-103.59	-60.17	-25.44	54.75	31.80	13.44
NH_3/BF_3	127.87	-94.54	-55.59	-22.56	54.74	32.19	13.07
C_2H_4/BF_3	9.17	-6.43	-1.57	-4.47	51.58	12.55	35.87
C_2H_2/BF_3	7.45	-5.67	-1.22	-3.81	53.03	11.38	35.59
H_2/BF_3	1.77	-1.03	-0.35	-1.22	39.68	13.36	46.95
H_2O/BF_3	75.24	-52.38	-29.82	-13.78	54.58	31.06	14.36
CH_3OH/BF_3	96.40	-66.35	-41.74	-18.32	52.49	33.02	14.50
$HCOOH/BF_3$ - I	111.98	-73.81	-52.10	-20.92	50.27	35.49	14.25
$HCOOH/BF_3$ - II	11.68	-9.12	-2.53	-4.83	55.35	15.38	29.28
CH_3COOH/BF_3 - I	121.80	-81.83	-58.14	-22.39	50.40	35.81	13.79
CH_3COOH/BF_3 - II	17.20	-12.95	-4.16	-6.72	54.36	17.44	28.20
HF/BF_3	6.58	-5.98	-1.44	-2.77	58.67	14.13	27.20
HCl/BF_3	4.97	-3.46	-0.86	-2.87	48.14	11.96	39.90
$(CH_3)_2NH/AlF_3$	68.28	-80.15	-30.64	-12.13	65.21	24.93	9.87
CH_3NH_2/AlF_3	64.52	-77.19	-29.07	-10.50	66.11	24.89	8.99
NH_3/AlF_3	59.28	-71.30	-27.28	-8.83	66.38	25.40	8.22
C_2H_4/AlF_3	35.67	-27.35	-19.49	-9.51	48.53	34.59	16.87
C_2H_2/AlF_3	33.06	-26.48	-18.08	-8.55	49.86	34.05	16.09
H_2/AlF_3	13.46	-7.04	-8.08	-3.71	37.36	42.92	19.72
H_2O/AlF_3	46.88	-52.17	-23.33	-7.34	62.98	28.16	8.86
CH_3OH/AlF_3	50.71	-56.51	-26.78	-8.76	61.39	29.09	9.52
$HCOOH/AlF_3$ - I	78.18	-75.25	-42.83	-12.83	57.48	32.72	9.80
$HCOOH/AlF_3$ - II	42.94	-40.65	-22.95	-8.50	56.38	31.83	11.79
CH_3COOH/AlF_3 - I	80.17	-80.02	-44.08	-13.05	58.34	32.14	9.51
CH_3COOH/AlF_3 - II	46.60	-45.67	-24.96	-9.81	56.78	31.03	12.19
HF/AlF_3	32.83	-30.53	-15.92	-5.73	58.50	30.51	10.99
HCl/AlF_3	25.66	-17.54	-15.23	-6.80	44.32	38.49	17.19
$(CH_3)_2NH/GaF_3$	96.27	-98.92	-32.00	-16.10	67.28	21.77	10.95
CH_3NH_2/GaF_3	90.70	-94.42	-30.01	-14.30	68.06	21.63	10.31
NH_3/GaF_3	83.62	-87.13	-27.99	-12.47	68.29	21.94	9.77
C_2H_4/GaF_3	57.43	-40.63	-23.30	-14.25	51.96	29.80	18.23
C_2H_2/GaF_3	49.88	-36.85	-20.18	-12.25	53.19	29.12	17.68
H_2/GaF_3	20.02	-10.01	-8.39	-5.20	42.41	35.57	22.02
H_2O/GaF_3	60.00	-57.93	-21.51	-9.73	64.96	24.12	10.92
CH_3OH/GaF_3	65.95	-63.56	-25.01	-11.46	63.54	25.01	11.46
$HCOOH/GaF_3$ - I	99.21	-86.05	-44.63	-16.25	58.56	30.38	11.06
$HCOOH/GaF_3$ - II	53.85	-44.86	-20.82	-10.82	58.64	27.22	14.14
CH_3COOH/GaF_3 - I	101.80	-91.16	-45.66	-16.52	59.45	29.78	10.77
CH_3COOH/GaF_3 - II	59.40	-50.94	-22.96	-12.56	58.92	26.56	14.52
HF/GaF_3	39.67	-32.69	-14.06	-7.28	60.50	26.03	13.48
HCl/GaF_3	35.86	-23.50	-15.48	-9.29	48.69	32.07	19.24
